# A predictive model for ethylene-mediated auxin and cytokinin patterning in the *Arabidopsis* root

**DOI:** 10.1016/j.xplc.2024.100886

**Published:** 2024-03-19

**Authors:** Simon Moore, George Jervis, Jennifer F. Topping, Chunli Chen, Junli Liu, Keith Lindsey

**Affiliations:** 1 Department of Biosciences, Durham University, South Road, Durham DH1 3LE, UK; 2 Hubei Hongshan Laboratory, College of Life Science and Technology, Huazhong Agricultural University, Wuhan 430070, China; 3 National Key Laboratory for Germplasm Innovation and Utilization for Fruit and Vegetable Horticultural Crops, Huazhong Agricultural University, Wuhan, Hubei 430070, China

**Keywords:** *Arabidopsis* root, auxin patterning, cytokinin patterning, ethylene signaling, *in silico* digital root, spatiotemporal modeling

## Abstract

The interaction between auxin and cytokinin is important in many aspects of plant development. Experimental measurements of both auxin and cytokinin concentration and reporter gene expression clearly show the coexistence of auxin and cytokinin concentration patterning in *Arabidopsis* root development. However, in the context of crosstalk among auxin, cytokinin, and ethylene, little is known about how auxin and cytokinin concentration patterns simultaneously emerge and how they regulate each other in the *Arabidopsis* root. This work utilizes a wide range of experimental observations to propose a mechanism for simultaneous patterning of auxin and cytokinin concentrations. In addition to revealing the regulatory relationships between auxin and cytokinin, this mechanism shows that ethylene signaling is an important factor in achieving simultaneous auxin and cytokinin patterning, while also predicting other experimental observations. Combining the mechanism with a realistic *in silico* root model reproduces experimental observations of both auxin and cytokinin patterning. Predictions made by the mechanism can be compared with a variety of experimental observations, including those obtained by our group and other independent experiments reported by other groups. Examples of these predictions include patterning of auxin biosynthesis rate, changes in PIN1 and PIN2 patterns in *pin3*,*4*,*7* mutants, changes in cytokinin patterning in the *pls* mutant, PLS patterning, and various trends in different mutants. This research reveals a plausible mechanism for simultaneous patterning of auxin and cytokinin concentrations in *Arabidopsis* root development and suggests a key role for ethylene pattern integration.

## Teaser

Using a wide range of experimental observations, this study proposes a mechanism for simultaneous patterning of auxin and cytokinin concentrations. This mechanism reveals that ethylene signaling is important in achieving simultaneous auxin and cytokinin patterning, while also predicting other experimental observations. These results illustrate the importance of hormonal crosstalk in patterning auxin, cytokinin, and ethylene concentrations in the *Arabidopsis* root.

## Introduction

A major challenge in plant developmental biology is understanding how development is coordinated by interacting hormones and genes. Plant hormones (Santner and Estelle, 2009) can act antagonistically or synergistically to regulate cell proliferation, elongation, and differentiation (Garay-Arroyo et al., 2012; Vanstraelen and Benkova, 2012). The importance of the interaction between auxin and cytokinin in root and shoot development and the maintenance of cell proliferation was shown in very early experiments on cultured tobacco callus (Skoog and Miller, 1957), where the ratio of cytokinin to auxin determined the developmental pathway. Although all hormones are involved in the regulation of root development, auxin and cytokinin play central roles in regulating meristem size and root growth (Perilli et al., 2012; Chandler and Werr 2015; Schaller et al., 2015; Friml 2021; Roychoudhry and Kepinski 2022).

Auxin and cytokinin cellular concentrations are a function of multiple factors, including biosynthesis (Zhao 2010; 2014; Jones and Ljung 2011; Ljung 2013; Casanova-Sáez et al., 2021), degradation (Jones and Ljung 2011; Ljung 2013; Casanova-Sáez et al., 2021), and conjugation (Ludwig-Müller, 2011). Importantly, both auxin and cytokinin concentrations display distinct patterns in the *Arabidopsis* root. Measurements of auxin concentration revealed the presence of IAA concentration gradients within the *Arabidopsis* root tip, with a distinct maximum in the organizing quiescent center (QC) of the root apex (Petersson et al., 2009). Measurements of cytokinin concentration revealed an intercellular cytokinin gradient in the primary root tip, with maximum levels in the lateral root cap, columella, columella initials, and QC cells (Antoniadi et al., 2015). These experimental data directly show that auxin and cytokinin concentration patterns coexist in the *Arabidopsis* root.

Many reporter-gene expression studies for both auxin and cytokinin are also consistent with the existence of auxin and cytokinin gradients in the *Arabidopsis* root (Isoda et al., 2021; Jedlickova et al., 2022). Response patterning, generated by reporter constructs based on various naturally occurring and synthetic promoters, includes imaging of *IAA2::GUS* and *DR5::GFP* (Grieneisen et al., 2007), *DII-VENUS* (Brunoud et al., 2012), and *R2D2* (Liao et al., 2015) for auxin and *ARR5::GUS* (Werner et al., 2003) and *TCSn::GFP* (Zürcher et al., 2013) for cytokinin. Because the relationship between hormone concentration and reporter-gene expression can be non-linear, the patterning of reporter-gene expression can differ from that of concentration. Moreover, expression patterning can vary between different response reporters, because, in addition to hormone concentrations, reporter response patterning also depends on other factors such as the sensitivity of the reporter promoter to the hormone. Moreover, reporter expression can also be influenced by multiple signaling pathways as shown in Liu et al. (2017). Nevertheless, patterning of reporter-gene expression for both auxin and cytokinin has been widely accepted as a proxy for auxin and cytokinin concentration patterns.

Measurements of both auxin and cytokinin concentrations and reporter gene expression clearly show the coexistence of auxin and cytokinin concentration patterning during *Arabidopsis* root development (Werner et al., 2003; Petersson et al., 2009; Brunoud et al., 2012; Zürcher et al., 2013; Antoniadi et al., 2015; Grieneisen et al., 2007; Liao et al., 2015). Moreover, a wide range of experimental data show that auxin, cytokinin, and ethylene form a complex crosstalk network. Despite progress in experimental studies, little is known about how auxin and cytokinin concentration patterns simultaneously emerge and how they regulate each other in the *Arabidopsis* root in the context of crosstalk among auxin, cytokinin, and ethylene. Importantly, although various experimental data accumulated over many years indicate that both auxin and cytokinin patterning play central roles in root development (Perilli et al., 2012; Chandler and Werr 2015; Schaller et al., 2015), an experimentally based mechanism for simultaneous auxin and cytokinin patterning is still elusive. For example, what is the mechanism for the emergence of auxin biosynthesis rate patterning, as experimentally measured in Petersson et al. (2009)? What is the mechanism for changes in PIN1 and PIN2 patterning in a variety of *pin* mutants, as observed by Blilou et al. (2005) and Omelyanchuk et al. (2016)? And what is the mechanism for changes in cytokinin patterning in the *pls* mutant reported by Casson et al. (2002)? Therefore, to further elucidate the mechanisms that drive root development, it is essential to better understand the complex multiple relationships among auxin and cytokinin and other developmentally critical hormones, proteins, and processes.

Auxin fluxes in the *Arabidopsis* root can be described by reverse fountain models (Petrasek and Friml, 2009). Auxin influx and efflux transporters play a key role in auxin patterning (Petrasek and Friml, 2009). Auxin patterning in the *Arabidopsis* root has been subjected to extensive research, in particular by combining experimental and modeling approaches (Grieneisen et al., 2007; Band et al., 2014; Moore et al., 2015; 2017; Rutten et al., 2022). Crosstalk between auxin and cytokinin has also been the subject of combined experimental and modeling studies. For example, Muraro et al. (2011, 2013, 2016) studied how cytokinin affects auxin-regulated gene expression and how meristem size is regulated by both auxin and cytokinin. Modeling of auxin and cytokinin crosstalk has also been used to elucidate root vascular patterning (De Rybel et al., 2014; Muraro et al., 2014; El-Showk et al., 2015; Mellor et al. 2017, 2019; Bagdassarian et al., 2023). In addition, how complex auxin and cytokinin crosstalk regulates cell-fate specification has also been modeled (Garcia-Gomez et al., 2017; 2020). However, in the context of crosstalk among auxin, cytokinin, and ethylene, little is known about how auxin and cytokinin concentration patterns regulate each other in the *Arabidopsis* root. We previously showed that, although auxin patterns can be correctly generated by integrating a hormonal crosstalk network with auxin transporters (Moore et al., 2015; 2017), the modeled cytokinin patterns are not in agreement with experimental observations (Moore et al., 2015; Liu et al., 2017). In this work, we propose an integrative mechanism for the simultaneous patterning of both auxin and cytokinin in the *Arabidopsis* root based on a wide range of experimental data from the literature. The mechanism for simultaneous auxin and cytokinin patterning in the *Arabidopsis* root is unraveled by integrating a range of experimental data and is validated by both our experimental data (such as PLS protein patterning and changes in cytokinin response patterning in the *pls* mutant) and independent experimental data (such as patterning of the rate of auxin biosynthesis and changes in PIN1 and PIN2 protein patterning in *pin3*, *pin4*, *pin7*, and *pin3*,*4*,*7* mutants).

## Results

### Interrogating and integrating biological knowledge to propose an integrative mechanism for simultaneous patterning of auxin and cytokinin in the *Arabidopsis* root

In developing a model to explain how auxin and cytokinin patterning can be generated, we first looked at relevant evidence from experimental studies. Nordstrom et al. (2004) proposed that auxin inhibits cytokinin biosynthesis and that cytokinin inhibits auxin biosynthesis in the whole seedling. Results indicated that different types of cytokinin (iP and Z types) were predominantly synthesized in either the shoot (Z) or the root (iP) and that, whereas biosynthesis of Z-type cytokinin was inhibited by auxin, biosynthesis of iP-type cytokinin was not inhibited and was even potentially promoted by application of auxin. Therefore, an additional conclusion from this paper could be that, whereas auxin inhibits cytokinin biosynthesis in the whole plant, it may not inhibit cytokinin biosynthesis in the root and could possibly promote it.

Additional studies suggest that cytokinin promotes auxin biosynthesis (Jones et al., 2010), auxin upregulates cytokinin biosynthesis through *SHY2* and *IPT5* genes (Dello Ioio et al., 2008), and auxin promotes cytokinin biosynthesis through TM05 and LOG4 (De Rybel et al., 2014). Jones et al. (2010) concluded that cytokinin promotes auxin biosynthesis in young developing tissues and that cytokinin inhibits its own biosynthesis through the induction of cytokinin oxidases (CKXs).

Cytokinin concentrations are determined by the balance among biosynthesis, degradation, and transport. Biosynthesis is regulated by rate-limiting steps involving the IPT group of enzymes, whereas irreversible cytokinin degradation occurs through the action of a set of CKXs (Werner et al., 2003; 2006). Cytokinin signaling acts through receptors at the plasma membrane and the endoplasmic reticulum (ER) and then through a phospho-relay cascade to activate a set of Type-B ARR transcription factors that target the Type-A ARRs which, although not transcription factors, act as inhibitors of Type-B ARRs (To et al., 2007). Therefore, within this initial pathway, cytokinin limits its own responses. Cytokinin is also self-regulated by the activity of CKX: increased cytokinin treatment initially increases CKX activity and then reduces it (Figure 4 in Chatfield and Armstrong, 1986).

A Type-B ARR of particular interest is ARR2, which appears to have unique properties, as phosphorylated ARR2 is rapidly degraded by the proteasome while other Type-B ARRs are not (Kim et al., 2012). Non-degradable ARR2 was found to increase cytokinin sensitivity and upregulate Type-A ARRs. Multiple ARR2 binding motifs found in the promoter regions of cytokinin-induced genes have led to the suggestion that ARR2 could act as a master regulator of cytokinin signaling responses (Hwang and Sheen, 2001).

ARR2 also links the cytokinin pathway with the ethylene pathway (Hass et al., 2004). ARR2 binds the *ERF1* promoter and upregulates *ERF1* expression. A stabilized phosphorylated (active) ARR2 showed an ethylene response in the absence of ethylene, even in the presence of AVG, an inhibitor of ethylene biosynthesis. Furthermore, the *arr2* null mutant has a reduced ethylene response that is rescued by expression of *ARR2* under the control of the *35S* promoter. There are also links in the opposite direction from the ethylene pathway to the cytokinin pathway (Hass et al., 2004) via ARR2. The ethylene receptor ETR1 appears to phosphorylate ARR2, because the ethylene sensitive *etr1-7* (Cancel and Larsen, 2002) loss-of-function mutant (low receptor activity and high downstream ethylene signaling) has reduced levels of phosphorylated ARR2 (Hass et al., 2004). It was concluded that an ETR1-dependent phospho-relay regulates ARR2 phosphorylation and activity (Hass et al., 2004). An additional link between the ethylene and cytokinin pathways is that EIN3 inhibits *ARR5*, a Type-A ARR commonly used in cytokinin reporter constructs (Shi et al., 2012; El-Showk et al., 2013).

There are also multiple links between the cytokinin and auxin pathways. Auxin upregulates *IPT* genes through SHY2 (Dello Ioio et al., 2008; Kushwah et al., 2011), and Type-B ARRs ARR1 and ARR12 in turn promote *SHY2* (Dello Ioio et al., 2008; El-Showk et al., 2013), which inhibits *ARF* in the auxin signaling pathway. Auxin also promotes the transcription of *AHP6*, an inhibitor of cytokinin signaling response (Bishopp et al., 2011).

ARR2 is suggested to be a central Type-B ARR within the cytokinin signaling pathway, with links to and from the ethylene pathway. Microarray analysis indicates that ARR2 promotes *CKX* expression and activity, as *CKX* mRNA is reduced by 2.9-fold in the *arr2* null mutant and increased by 14.1-fold with stabilized activated ARR2, which cannot be degraded and mimics phosphorylation (Hass et al., 2004). Therefore, activity of the ethylene receptor ETR1 appears to be able to regulate cytokinin concentrations and response through ARR2 and CKX by phosphorylating ARR2 and increasing its activity (Hass et al., 2004). As such, it is proposed that active ETR1 receptors (in the absence of ethylene) result in ARR2 phosphorylation and increased ARR2 activity, which in turn results in increased CKX activity and reduced cytokinin. In the presence of ethylene, ETR1 activity is reduced, which decreases ARR2 phosphorylation and activity and so reduces CKX activity and increases cytokinin concentrations. This is consistent with experimental results that showed an increase in cytokinin concentration in the ethylene hyper-signaling *pls* mutant compared with the wild type (Liu et al., 2010).

**Figure 1:**
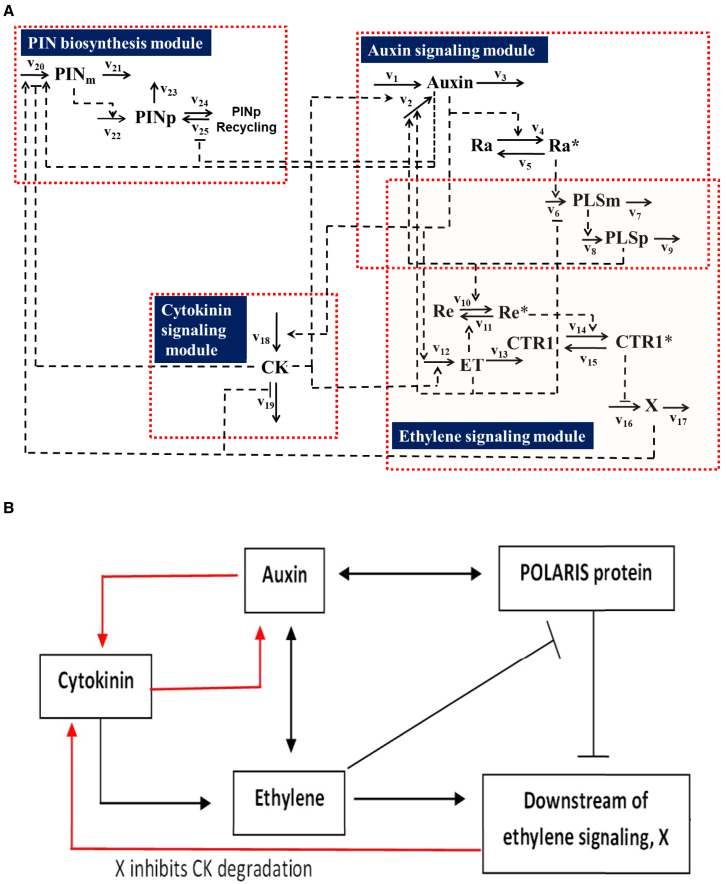
An integrative mechanism for simultaneous auxin and cytokinin patterning in *Arabidopsis* root development. Auxin metabolism in each cell is regulated by ethylene and cytokinin signaling. Auxin transport within a cell is due to diffusion, and its transport between cells is predominantly facilitated by the functions of PIN and AUX1, LAX2/3 transporters. Cytokinin metabolism is regulated by auxin and ethylene, and cytokinin is transported via diffusion. Ethylene metabolism is regulated by auxin and cytokinin, and ethylene is transported via diffusion. The POLARIS protein regulates ethylene signaling by interacting with its receptors. The proposed mechanism integrates metabolism and transport of auxin, cytokinin, and ethylene into an integrative system, and the patterning of the three hormones is mutually regulated. **(A)** Detailed network for mutual regulation of auxin, cytokinin, and ethylene; the network is extracted from the more comprehensive network in Liu et al. (2017). **(B)** Schematic description of mutual regulation among auxin, cytokinin, and ethylene. This is a simplified summary of the detailed network (Figure 1A), with red lines highlighting links based on additional biological evidence that is analyzed in detail in this research. → indicates activation; –| indicates inhibition. Connections marked with red lines in **(B)** highlight the novel regulatory relationships explored in this research. Connections marked with black lines in **(B)** are regulatory relationships examined previously (Moore et al., 2015; 2017; Liu et al., 2017). All connections are based on either experimental data from our lab or established biological evidence from the literature. Auxin, auxin hormone; ET, ethylene; CK, cytokinin; PINm, PIN mRNA; PINp, PIN protein; PLSm, POLARIS mRNA; PLSp, POLARIS protein; X, downstream ethylene signaling; Ra∗, active form of auxin receptor; Ra, inactive form of auxin receptor; Re∗, active form of the ethylene receptor ETR1; Re, inactive form of the ethylene receptor ETR1; CTR1∗, active form of CTR1; CTR1, inactive form of CTR1.

These lines of evidence indicate that ethylene responses positively regulate cytokinin concentration by inhibiting CKX activity. In previous studies on auxin, cytokinin, and ethylene crosstalk in the *Arabidopsis* root (Liu et al., 2010; 2013; Moore et al., 2015, 2017), the regulation of cytokinin concentration by ethylene signaling was not studied. In previous work (Moore et al., 2015), even after cytokinin biosynthesis was restricted to the vascular cylinder, the modeled cytokinin patterning was still significantly different from experimental observations (Werner et al., 2003). Previous studies were only able to reproduce auxin patterning, and an experimentally based mechanism for the simultaneous emergence of auxin and cytokinin patterning in the *Arabidopsis* root remained elusive. Here, we show that the biological evidence discussed above is vital to explaining the simultaneous patterning of auxin and cytokinin, while also allowing model predictions to match other experimental observations. Figure 1A summarizes the mechanism in detail, and Figure 1B shows a simplified form in which red lines highlight the biological evidence discussed above. Specifically, this mechanism indicates that cytokinin promotes auxin biosynthesis, auxin promotes cytokinin biosynthesis, and ethylene signaling inhibits cytokinin degradation, thus promoting cytokinin accumulation. As demonstrated below, combining such a mechanism with cell–cell communications simultaneously generates auxin and cytokinin patterning and also makes predictions that match independent experimental observations.

### Relationships among auxin, cytokinin, and ethylene in a homogenous cell according to this mechanism

**Figure 2:**
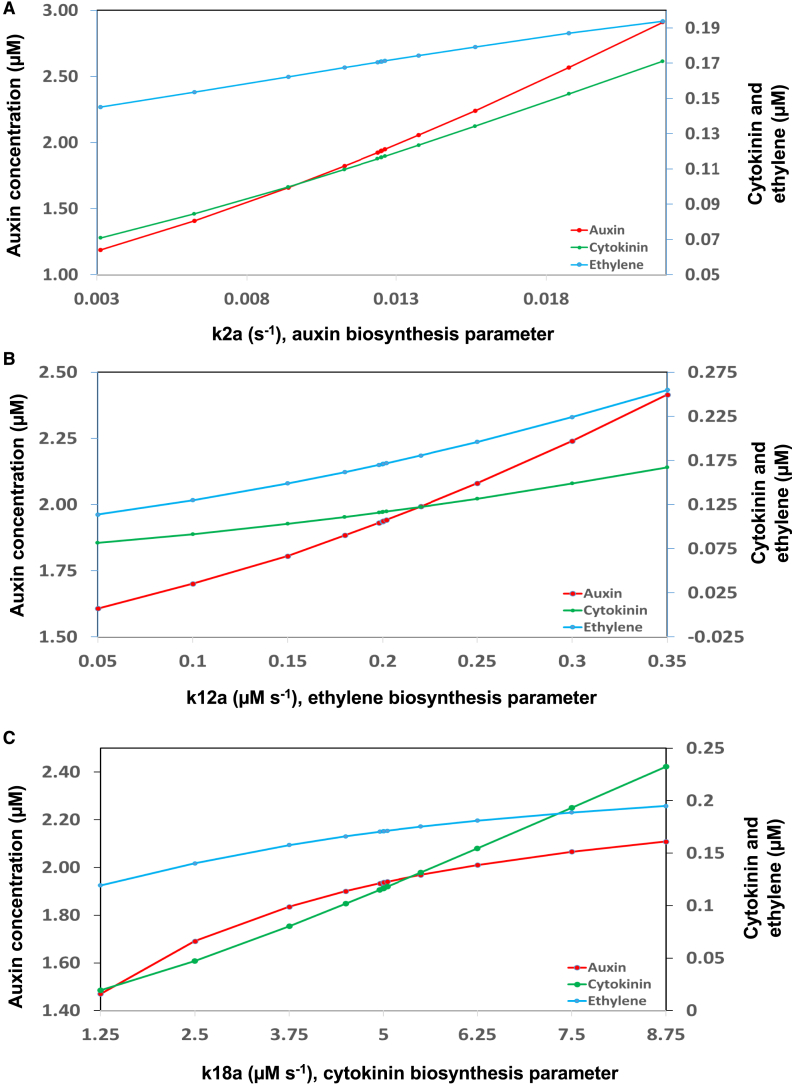
Relationships among auxin, cytokinin, and ethylene in a homogenous cell. **(A)** Effects of changing auxin biosynthetic rate on auxin, cytokinin, and ethylene concentrations. **(B)** Effects of changing ethylene biosynthetic rate on auxin, cytokinin, and ethylene concentrations. **(C)** Effects of changing cytokinin biosynthetic rate on auxin, cytokinin, and ethylene concentrations.

The mechanism shown in Figure 1A and 1B describes how auxin, cytokinin, and ethylene mutually promote each other. For a cell without communications with other cells, Figure 2 shows that increasing auxin, cytokinin, or ethylene biosynthesis rate always simultaneously enhances the concentrations of all three hormones. For example, increasing or decreasing the key parameter for auxin biosynthesis by 1% from its wild-type value (Figure 2A) results in a similar increase or decrease in both auxin and cytokinin concentrations by ∼0.6%, whereas ethylene concentration increases or decreases by ∼0.2%. Supplemental Figure 1 shows an example of simultaneously enhancing the concentrations of auxin, cytokinin, and ethylene by increasing the rate of auxin biosynthesis in homogenous cells of the root.

### The mechanism reproduces experimental observations of both auxin and cytokinin patterning in a realistic *in silico* root

The mechanism described in Figure 1 is a hormonal crosstalk network for auxin, ethylene, and cytokinin extracted from a more complex auxin, ethylene, and cytokinin network in *Arabidopsis* root development (Liu et al., 2017). Investigating patterning requires the combination of a crosstalk network with a realistic digital root structure. A method to generate a realistic two-dimensional digital root was previously developed and described in detail (Moore et al., 2017). The digital *in silico* root, as summarized in Figure 1 in Moore et al. (2017), is derived from experimental imaging and contains actual cell geometry and multicellular root organization that enable the study of cell–cell communication (Band et al., 2014). In the *in silico* root, each cell contains two spatial identities: the cytosol, and the plasma membrane and cell wall, which are specific to each cell. For simplicity, adjacent plasma membrane and cell wall entities for the same cell are represented by a single identity that contains both cell wall and plasma membrane properties. The *in silico* root also includes the extracellular space but does not include any subcellular structures.

The regulation and placement of the auxin influx and efflux carriers (PIN1,2,3,4,7 and AUX1, LAX2,3, and ABCB) within the *in silico* root are based on experimental data, as described previously (Moore et al., 2015; 2017). PIN1 and PIN2 carrier levels are regulated by the three hormones (Figure 1A). The rate at which cytosolic PIN1 or PIN2 protein is placed at, and removed from, the plasma membrane was selected so that their polarity in the *in silico* root was similar to experimentally observed polarity (Moore et al., 2015; 2017). Other auxin carriers are prescribed on the basis of experimental data because there are insufficient experimental data to establish how their levels and polarity are regulated by the three hormones (Moore et al., 2017). PIN3, PIN4, and PIN7 efflux carriers have prescribed concentration levels and polar localizations at selected cell faces based on experimental imaging described in the literature (Blilou et al., 2005). The non-polar auxin influx carriers AUX1, LAX2, and LAX3 also have prescribed localizations and levels based on experimental imaging (Band et al., 2014). The relative concentrations of PIN3, PIN4, PIN7, AUX1, LAX2, and LAX3 carriers are adjusted to generate wild-type auxin patterning. Because the ABCB family of auxin carriers can reversibly redirect auxin flux, the role of ABCB carriers in auxin transport has been implicitly incorporated into the non-polar base levels of PIN and AUX1/LAX activity to simplify modeling analysis.

**Figure 3:**
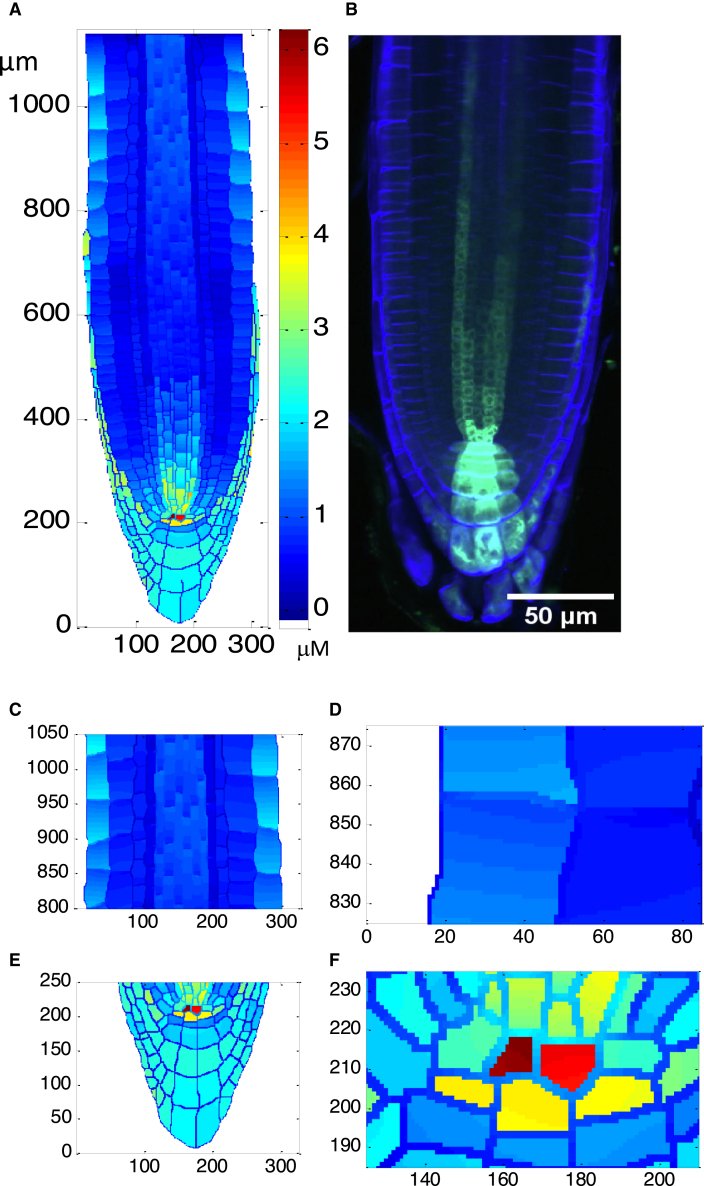
Modeled auxin concentration patterning. Modeled auxin patterning **(A** and **C–F)** is in good agreement with both IAA concentration distribution measured experimentally (supplemental Figure 2A; Figure 3 in Petersson et al., 2009) and auxin response patterning shown as DR5:GFP fluorescence **(B)**. In the longitudinal direction, the meristem is at ∼200–650 μm, the transition zone (TZ) at ∼650–750 μm, and the elongation zone at ∼750–1200 μm. Equations and parameter values are included in supplemental Tables S1–S3.

**Figure 4:**
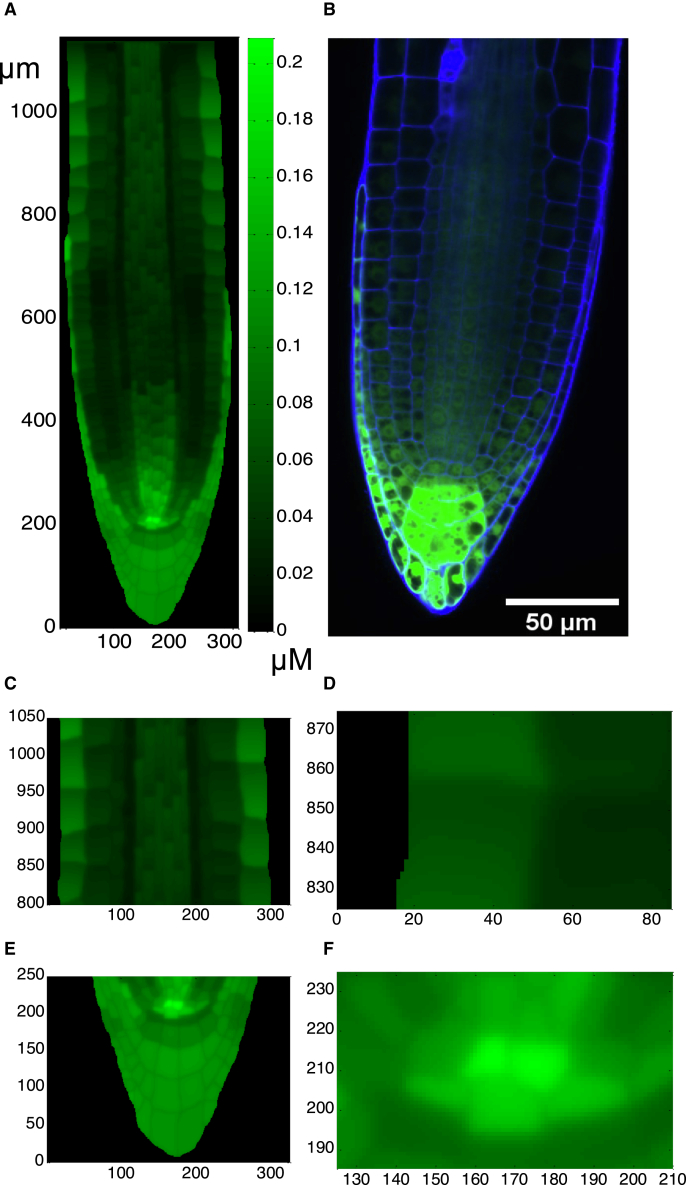
Modeled cytokinin concentration patterning. Modeled cytokinin concentration patterning **(A** and **C–F)** is in good agreement with both cytokinin concentration distribution measured experimentally (supplemental Figure 3A; Figure 5 in Antoniadi et al., 2015) and cytokinin response patterning shown as ARR5:GFP fluorescence **(B)**. Equations and parameter values are included in supplemental Table 1.

Where available, parameter values from the literature have been used (Moore et al., 2015; 2017). Because it is unknown whether biological information accumulated in the literature is capable of simultaneously generating auxin and cytokinin patterning in the *Arabidopsis* root, we have adjusted the unknown parameters and examined the patterning of both auxin and cytokinin to test the relationship between the known signaling interactions described above and the simultaneous patterning of auxin and cytokinin. Figures 3 and 4 show that the mechanism described in Figure 1, coupled with the realistic *in silico* root, can simultaneously generate auxin and cytokinin patterning that is in general agreement with experimental results. This indicates that the biological information accumulated in the literature is sufficient to describe a mechanism that simultaneously generates auxin and cytokinin patterning in the *Arabidopsis* root.

Figure 3A and 3B show that the auxin concentration patterning generated by this mechanism is similar to auxin response patterning observed using DR5-GFP fluorescence. Figure 3A and 3C–3F display the auxin concentration patterning generated by this mechanism, showing patterning in the root tip (Figure 3A) and progressive enlargements in the elongation zone (EZ) (Figure 3C and 3D) and QC region (Figure 3E and 3F). A pronounced auxin maximum occurs in the QC region (Figure 3A, 3E, and 3F), with relatively high auxin levels in the columella and the root cap (Figure 3A and 3E). Auxin concentration declines from the QC maximum in the cell files above the initials and proximally up the vascular cylinder (Figure 3A and 3E). Interestingly, there is a clear increase in auxin concentration in the epidermis starting in the transition zone (TZ) and moving into the EZ (Figure 3A and 3C) and a similar but less obvious increase in the cortex (Figure 3A). Figure 3C and 3D shows the existence of predicted auxin gradients within individual cells, with auxin declining at the proximal boundary of the epidermal cells in the EZ where the PIN efflux carriers remove auxin from the cell, then increasing at the distal boundary of the neighboring shootward cell owing to the action of the auxin influx carriers. Auxin gradients may also emerge in the extracellular space owing to auxin diffusion (Figure 3D and 3F).

Modeled auxin concentration patterning (Figure 3A and 3C–3F) is in agreement with various experimental observations, including IAA distribution (supplemental Figure 2A; Figure 3 in Petersson et al., 2009), auxin response patterning revealed by *DR5::GFP* and *IAA2::GUS* (supplemental Figure 2B and 2C; Figures 3 and 4 in Grieneisen et al., 2007), and relative auxin levels based on the DII-VENUS inverse auxin response reporter (supplemental Figure 2D; Figure 2 in Brunoud et al., 2012). The different experimental observations give somewhat differing results for auxin patterning. The relative IAA distribution, based on cell sorting and mass spectrometry of various *Arabidopsis* lines, shows a distinct auxin maximum in the QC region and higher auxin levels in the lateral root cap, cortex, endodermis, and vascular cylinder compared with the columella and epidermis (supplemental Figure 2A; Figure 3 in Petersson et al., 2009). However, apart from the distinct differences between these regions, the relative IAA distribution does not provide much additional information on auxin gradients. The *DR5::GFP* auxin response reporter (supplemental Figure 2B; Figure 3 in Grieneisen et al., 2007) reveals a high auxin response in the QC region and the proximal region of the columella, with the signal quickly declining in a shootward direction along the center line of the vascular cylinder. There is no indication of relatively high auxin response in the lateral root cap or of an increase in the epidermis in the EZ. *In silico* results are in better agreement with the *IAA2::GUS* auxin reporter (supplemental Figure 2C; Figure 4 in Grieneisen et al., 2007), with a high auxin response in the QC region, columella, and lateral root cap and a proximally declining signal in the vascular cylinder. However, a noticeable difference between the *in silico* results and the experimental data based on the *IAA2::GUS* reporter is that the reporter does not indicate a signal increase in the epidermis of the EZ. The auxin response image derived from the DII-VENUS auxin reporter (supplemental Figure 2D; Figure 2 in Brunoud et al., 2012), which gives an inverse auxin response signal, shows a maximum auxin response in the QC, a high auxin response in the columella and lateral root cap, and a reduced response moving shootward along the vascular cylinder. In the epidermis, the response declines proximal to the initials and then starts to increase again at the beginning of the TZ, which is approximately longitudinal position 650 in Figure 3. *In silico* patterning is in good agreement with results generated by the DII-VENUS reporter (supplemental Figure 2D; Figure 2 in Brunoud et al., 2012). However, this epidermal patterning trend is not observable using *DR5::GFP* (Figure 3B; supplemental Figure 2B). Following Brunoud et al. (2012), a possible explanation is that responses of these reporters can differ, as the *DR5* auxin response is also influenced by multiple signaling pathways.

Many features of *in silico* cytokinin concentration patterning (Figure 4A and 4C–4F) are similar to those of *in silico* auxin patterning (Figure 3A and 3C–3F). Cytokinin displays a maximum relative concentration in the QC region (but not to the same degree as auxin), with high concentrations in the columella and lateral root cap and with gradients similar to those of auxin in the vascular cylinder and epidermis.

Key features of the modeled cytokinin concentration patterning agree with experimental observations using ARR5-GFP fluorescence (Figure 4B) and other experimental observations as described below. The relative cytokinin concentrations measured by cell sorting and mass spectrometry (supplemental Figure 3A; Figure 5 in Antoniadi et al., 2015) indicate high cytokinin concentrations in the QC region, columella, and lateral root cap, medium concentrations in the vascular cylinder and in the epidermis and cortex of the TZ and EZ, and lower concentrations in the endodermis. The *in silico* concentration patterning (Figure 4A and 4C–4F) is very similar to these experimental observations. Cytokinin response was also measured using the *ARR5::GUS* reporter (supplemental Figure 3B; Figure 3 in Werner et al., 2003) or the *TCSn::GFP* reporter (supplemental Figure 3C; Figure 4 in Zürcher et al., 2013).

One marked difference between the experimental results for relative cytokinin concentration measurements (supplemental Figure 3A; Figure 5 in Antoniadi et al., 2015) and cytokinin response observations (Figure 4B; supplemental Figure 3B; Figure 3 in Werner et al., 2003; supplemental Figure 3C; Figure 4 in Zürcher et al., 2013) occurs in the QC region. Although a high cytokinin concentration is measured in the QC by Antoniadi et al. (2015), a high cytokinin response is observed in the initials just proximal to the QC (Figure 4B; supplemental Figure 3B; Figure 3 in Werner et al., 2003; supplemental Figure 3C; Figure 4 in Zürcher et al., 2013). The modeled concentration patterning (Figure 4A) more closely matches the measured cytokinin concentration patterning in the QC (supplemental Figure 3A; Figure 5 in Antoniadi et al., 2015). A possible explanation for the difference between these experimental results is that cytokinin response is suppressed by the very high auxin concentration in the QC via AHP6 signaling (Bishopp et al., 2011; Liu et al., 2017). On the other hand, Figure 4A and 4E also shows that cytokinin concentration in a row of cells below the QC is noticeably lower. This is inconsistent with the measured cytokinin concentration patterning in these cells (supplemental Figure 3A; Figure 5 in Antoniadi et al., 2015). Thus, how AHP6 signaling regulates the cytokinin response in these cells remains to be elucidated, as the modeled auxin concentration in these cells is relatively low (Figure 3).

### Modeling predictions match a variety of experimental observations

The above results demonstrate that simultaneous patterning of both auxin and cytokinin in the *Arabidopsis* root (Figures 3 and 4) can emerge from a model based on a wide range of biological data. A further question is whether the mechanism can predict independent experimental observations. To predict different experimental outputs, we integrated the mechanism with the realistic digital root and parameters for reproducing simultaneous patterning of both auxin and cytokinin. This allows *in silico* predictions to be compared with a variety of experimental observations generated by our group and independently by other groups, as detailed below.

#### Auxin response trends in cell files above the initials

**Figure 5:**
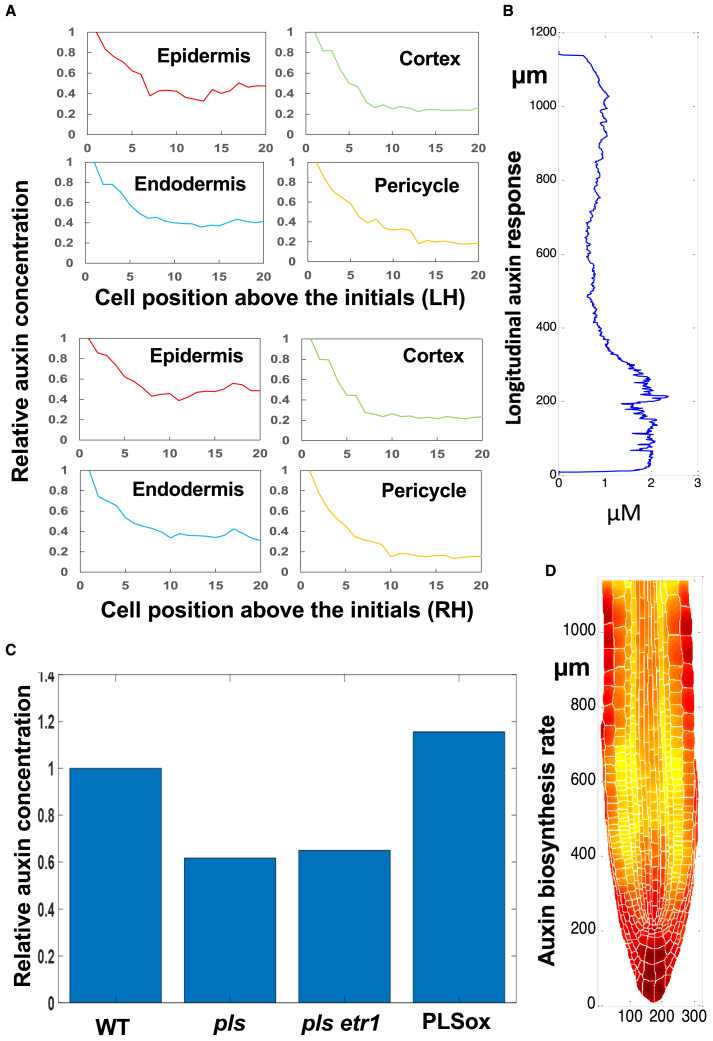
Spatiotemporal modeling predicts experimental observations of auxin concentration, patterning, and biosynthetic rate reported in the literature. **(A)** Auxin response distribution along cell position from the initials of different cell types (similar to supplemental Figures 4 and 6 in Liao et al., 2015). **(B)** Minimum auxin response distribution in the TZ (similar to supplemental Figure 5; Figure 3 in Di Mambro et al., 2017). **(C)** Average auxin concentration in different mutants (similar to supplemental Figure 6; Figure 4 in Chilley et al., 2006). **(D)** Auxin biosynthetic rate distribution (similar to supplemental Figure 7; Figure 5 in Petersson et al., 2009). In the longitudinal direction, the meristem is at ∼200–650 μm, the TZ at ∼650–750 μm, and the elongation zone at ∼750–1200 μm.

Relative auxin concentration trends in the epidermis, cortex, and endodermis cell files above the initials were experimentally established using the R2D2 reporter (supplemental Figures 4A and 7 in Liao et al., 2015). Using the *in silico* wild type, auxin concentration was calculated at all grid points in the digital root. The average auxin concentration in each cell was then calculated by averaging all grid points within the cell. The computed auxin trends for the epidermis, cortex, and endodermis cell files above the initials (Figure 5A) closely matched experimental results, as shown by superimposing the *in silico* results onto the experimental graph (supplemental Figure 4B–4E). Specifically, in all four cell layers, a significant decrease in auxin concentration is observed in the cells immediately above the initials, then the reduction in auxin concentration becomes much slower. In the epidermis, after the initial decrease, auxin concentration increases slightly for the cells further above the initial. In the cortex and the pericycle, the overall decrease in relative auxin concentration, in both experimental results and modeling results, is somewhat greater than that observed in other cell layers. This demonstrates that the model is able to predict the pattern of auxin concentration for different tissues. However, when the four cell files are compared with each other, supplemental Figure 4F shows that, for the cells near the initials, the level of the average auxin concentration does not follow a clear order. This is in contrast to experimental observations (supplemental Figure 4A), in which the average auxin concentration follows a clear order and sequentially decreases from the epidermis, endodermis, and cortex to the pericycle. For cells further above the initials, the average auxin concentration follows a clear order that agrees with experimental observations. This indicates a limitation of the model in quantitatively predicting the order of average auxin concentration among some cells of the four cell files.

#### Auxin response minimum in the TZ

Experimental results indicate that a minimum in the auxin response along the root axis (supplemental Figure 5A; Figure 3 in Di Mambro et al., 2017) occurs at the boundary of the proximal meristem and the distal TZ and that this minimum triggers a key developmental switch between cell division and cell differentiation (Di Mambro et al., 2017). Using the *in silico* wild type, we calculated a longitudinal auxin concentration profile (Figure 5B) by progressively averaging auxin concentrations along the axis of the root (Figure 2A). The *in silico* results (Figure 5B) display an auxin concentration minimum in the distal TZ, similar to experimental observations (supplemental Figure 5A; Figure 3 in Di Mambro et al., 2017). Specifically, the longitudinal auxin concentration profile (Figure 5B) shows that auxin concentration decreases quickly from the QC, remains approximately unchanged until above the TZ, then increases in the EZ. This result demonstrates that, after both auxin and cytokinin patterning are fitted to experimental observations, an auxin concentration minimum intrinsically emerges in the distal TZ (supplemental Figure 5B).

#### Trends in verage auxin concentration in the WT, *pls* mutant, *pls etr1* double mutant, and PLSox

Experimental data have demonstrated that up- or downregulation of the *PLS* gene or the ethylene receptor protein ETR1 alters ethylene signaling responses (Casson et al., 2002; Chilley et al., 2006; Liu et al., 2010). In the *etr1* mutant, the ethylene signaling response is upregulated. Figure 5C shows that *in silico* predictions of the trend in average auxin concentration for the *pls* mutant, *pls etr1* double mutant, and *PLS*-overexpression transgenic line (*PLSox*) are in general agreement with experimental observations (supplemental Figure 6A; Figure 4C in Chilley et al., 2006). The experimentally measured auxin concentration is lower in the *pls mutant* than in the wild type; higher in the *pls etr1 double mutant* than in the *pls* mutant but still slightly lower than in the wild type; and higher in the PLS-overexpressing seedlings than in the wild type (supplemental Figure 6A; Figure 4C in Chilley et al., 2006). Modeled auxin concentration trends were superimposed over the experimental results (supplemental Figure 6B). The modeled trends were in general agreement with experimental observations, although modeled auxin concentration was markedly lower in the *pls etr1 double mutant* than in its experimental counterpart. PLS is a protein that interacts with ethylene receptors (Casson et al., 2002; Chilley et al., 2006; Liu et al., 2010; Mudge et al., 2023), and manipulation of PLS activity affects ethylene signaling (Chilley et al., 2006; Liu et al., 2010). Therefore, when both auxin and cytokinin patterning are fitted to experimental observations according to the proposed mechanism (Figure 1), the effects of genetic manipulation of ethylene signaling on average auxin concentration in the root can also be predicted.

#### Patterning the rate of auxin biosynthesis

The rate of auxin biosynthesis also demonstrates patterning in the root tip (supplemental Figure 7; Figure 5 in Petersson et al., 2009). In the *in silico* wild-type simulation, the computed patterning of auxin biosynthetic rate shows high levels of biosynthesis in the columella and the QC region, lower levels in the TZ, and an increase in the epidermis, cortex, and central vascular cylinder of the EZ. There is a close match between experimental (supplemental Figure 7; Figure 5 in Petersson et al., 2009) and predicted auxin biosynthesis rate patterning (Figure 5D). Thus, the proposed mechanism is able to predict not only important features of auxin concentration patterning but also patterning of auxin biosynthetic rate. Therefore, auxin patterning, transport, and metabolism are elucidated as an integrated system in this work.

#### Trends in average concentration of PIN1 and PIN2 proteins in the WT, PLSox, *pls* and *etr1* mutants, and *pls etr1* double mutant

**Figure 6:**
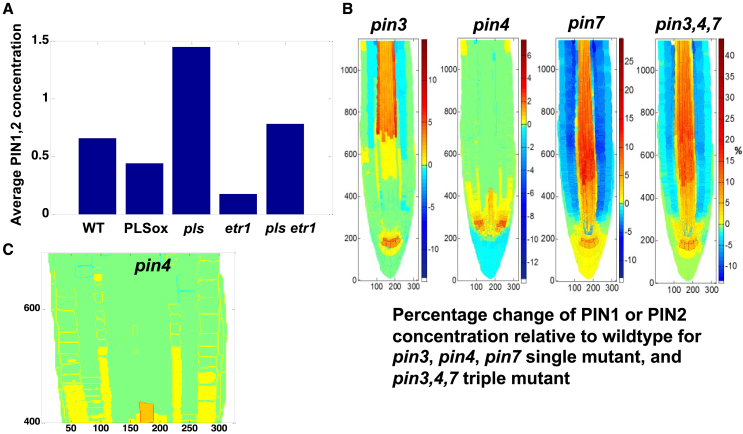
Spatiotemporal modeling predicts changes in PIN1 and PIN2 level and patterning in various mutants. **(A)** Modeled changes in PIN1 and PIN2 levels in various mutants (similar to supplemental Figure 8; Figure 1B in Liu et al., 2013). **(B)** Modeled changes in PIN1 and PIN2 patterning in *pin3*, *pin4*, and *pin7* single mutants (similar to supplemental Figure 9A; PIN1 patterning change in Figure 6 in Omelyanchuk et al., 2016) and in *pin3,4,7* triple mutants (similar to supplemental Figure 9B; PIN2 patterning change in Figure 1 in Blilou et al., 2005). **(C)** Enlargement of the *pin4* mutant in **(B)**.

Immunolocalization experiments (supplemental Figure 8; Figure 1B in Liu et al., 2013) showed that PIN1 and PIN2 protein levels are higher in the *pls* mutant and lower in PLSox than in the wild type; PIN1 and PIN2 levels are lower in the ethylene-insensitive *etr1 mutant* than in the wild type; and the *pls etr1* double mutant exhibits reduced PIN1 and PIN2 levels compared with *pls* and marginally lower levels than the wild type. The *in silico* trends for these mutants (Figure 6A) are similar to experimental observations, demonstrating that the proposed mechanism is able to predict changes in PIN1 and PIN2 levels and therefore predict how genetic manipulations alter the average concentrations of auxin transporters in the root.

#### Predicted changes in PIN1 and PIN2 concentrations in *pin3*, *pin4*, and *pin7* mutants

So far, we have demonstrated that the *in silico* wild-type simulation is able to predict many features of auxin patterning, as well as trends in auxin, PIN1, and PIN2 levels in various mutants. A further question is whether changes in PIN1 and PIN2 patterning can also be predicted.

Production and degradation of PIN1 and PIN2 in a cell are assumed to follow the same mechanism based on experimental observations as analyzed previously (Liu et al., 2013; Moore et al., 2015; 2017). However, PIN1 and PIN2 are distinguished by their polarity and localization in different parts of the root tip.

Figure 6B shows *in silico* predictions for the percentage change in PIN1 or PIN2 concentration relative to that of the WT for the *pin3*, *pin4*, and *pin7* single mutants and the *pin3pin4pin7* triple mutant. Figure 6C is an enlargement of the proximal vasculature for the *pin4* single mutant image in Figure 6B. Modeled changes in PIN1 patterning for the *pin3*, *pin4*, and *pin7* single mutants (Figure 6B and 6C) are similar to experimental observations (supplemental Figure 9A; Figure 6 in Omelyanchuk et al., 2016). In particular, the region of PIN1 expression extends shootward up the vasculature in these mutants (supplemental Figure 9A; Figure 6 in Omelyanchuk et al., 2016).

The modeled changes in PIN1 concentration of the *pin3* mutant versus the wild type (Figure 6B) show a significant increase in the proximal vasculature and the columella. This is consistent with experimentally observed PIN1 expression in the same mutant (supplemental Figure 9A; Figure 6 in Omelyanchuk et al., 2016).

Moreover, the modeled PIN1 concentrations in the *pin4* mutant show slight increases at the plasma membrane in the proximal vasculature (Figure 6B), consistent with experimental observations of the *pin4* mutant (supplemental Figure 9A; Figure 6 in Omelyanchuk et al., 2016).

The modeled change in PIN1 patterning in the *pin7* mutant (Figure 6B) is also similar to experimental observations (supplemental Figure 9A; Figure 6 in Omelyanchuk et al., 2016), in which an increase in *PIN1* concentration can be seen in the proximal vasculature of the *pin7* mutant.

Thus, the integrative mechanism for simultaneous auxin and cytokinin patterning is able to correctly predict changes in PIN1 patterning in *pin3*, *pin4*, and *pin7* mutants.

Experimental observations also show changes in PIN2 patterning in the *pin3pin4pin7* triple mutant. A clear increase in PIN2 level in the vasculature emerges (supplemental Figure 9B; Figure 1 in Blilou et al., 2005). Modeled changes in PIN2 patterning for the *pin3pin4pin7* triple mutant predict a significant increase in PIN2 concentrations in the vasculature (Figure 6B), consistent with experimental observations (supplemental Figure 9B; Figure 1 in Blilou et al., 2005).

These results indicate that the integrative mechanism proposed in this research predicts the main features of patterning changes in PIN1 and PIN2 concentrations in *pin3*, *pin4*, and *pin7* mutants and thus reveals how PIN1 and PIN2 patterning changes in single and triple *pin* mutants.

#### Cytokinin patterning and concentration changes in the *pls* mutant

Figures 5 and 6 demonstrate that the mechanism described in Figure 1 is able to predict patterning and/or trends in auxin concentration, auxin biosynthetic rate, and PIN1,2. The mechanism can also predict patterning and concentration changes in cytokinin in the *pls* mutant.

**Figure 7:**
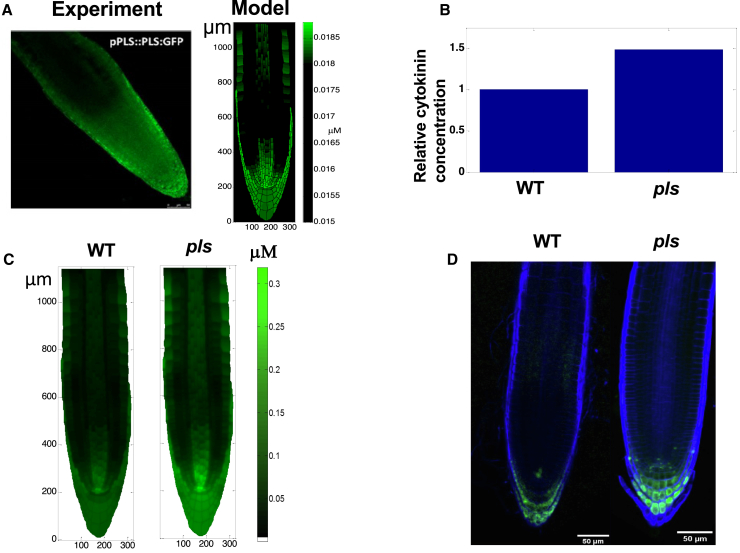
Modeled changes in cytokinin concentration and patterning in the *pls* mutant predict experimental observations. **(A)** Modeled PLS protein distribution (right panel) and measured PLS GFP fluorescence (left panel). The actual concentration of PLS protein has not been experimentally quantified, and therefore the color of the experimental image indicates the relative change in the level of PLS protein. **(B)** Modeled change in cytokinin concentration in the *pls* mutant relative to the wild type (similar to Table 1 in Liu et al., 2010). **(C)** Modeled change in cytokinin concentration in the *pls* mutant. (**D**) TCSn:GFP fluorescence in the wild type and *pls* mutant.

First, auxin, cytokinin, and ethylene all regulate gene expression (Weijers and Wagner 2016; Kieber and Schaller 2018; Binder 2020). *PLS* gene expression is regulated by both auxin and ethylene (Chilley et al., 2006; Liu et al., 2010). The modeled patterning of *PLS* expression predicts measured proPLS::PLS:GFP fluorescence (Figure 7A). Modeled PLS levels are high in the columella, lateral root cap, and QC region, consistent with experimental observations (Figure 7A). This demonstrates that the mechanism proposed in this research is also able to predict gene expression patterning.

PLS protein has a role in regulating ethylene signaling (Chilley et al., 2006; Liu et al., 2010), which in turn regulates auxin and cytokinin concentrations and signaling (Figure 1). Thus, an important question is how PLS patterning regulates the concentration and patterning of other components in Figure 1.

Figure 7B predicts that modeled cytokinin concentration is 1.48-fold higher in the *pls mutant* relative to the wild type. This compares favorably with experimental results (Table 1 in Liu et al., 2010), which show a median fold increase of 1.42. In addition, modeling reveals that the cytokinin concentration in *pls* shows a significant increase in the columella, lateral root cap, and QC region (Figure 7C), which is similar to experimental observations using TCSn:GFP fluorescence (Figure 7D). Thus, the effects of the *pls* mutant on the concentration and patterning of cytokinin are correctly predicted by the mechanism proposed in this research (Figure 1).

In addition, supplemental Figure 10 predicts that ethylene concentration in the *pls* mutant is the same as that in the wild type. This is again consistent with experimental observations (Chilley et al., 2006).

### Modeling reveals the regulatory relationships in auxin and cytokinin concentration and patterning

So far, we have demonstrated that the model based on the mechanism in Figure 1 is able to not only generate simultaneous patterning of both auxin and cytokinin but also predict a variety of experimental observations. Here, we show that the model can generate insights into the role of regulation in auxin and cytokinin patterning.

#### Auxin influx and efflux transporters are the key driver for auxin patterning

Supplemental Figure 11 shows that auxin patterning still emerges when auxin biosynthesis is not regulated by cytokinin or ethylene, with all auxin transporters being fixed as those in the wild type. This indicates that auxin influx and efflux transporters are the key players for generation of auxin patterning. Moreover, both cytokinin and ethylene patterning still emerge. However, supplemental Figure 11 also reveals that the percentage change in the concentrations of auxin, cytokinin, and ethylene are different in different cells and therefore that the regulation of auxin biosynthesis plays a fine-tuning role in patterning. In the absence of auxin biosynthesis regulation, supplemental Figures 12–14 show that predictions of the trends in different mutants are affected (supplemental Figures 12C, 12D, 13A, and 14B). In particular, the patterning of auxin biosynthesis rate can no longer be predicted (supplemental Figure 12D). In addition, supplemental Figure 11 shows that the concentrations of auxin, cytokinin, and ethylene are higher in the epidermis than in the cortex. This is consistent with the mutual positive regulation of auxin, cytokinin, and ethylene (Figures 1 and 2; supplemental Figure 1). This high concentration in the epidermis still exists when the regulation of auxin biosynthesis by ethylene and cytokinin is removed, but it is generally less obvious, as the percentage reduction relative to the wild type is relatively large (supplemental Figure 11). A high concentration in the epidermis was also modeled previously (Band et al., 2014) in the absence of regulation in auxin biosynthesis. Therefore, regulation of auxin biosynthesis plays a role in fine-tuning the high auxin concentration in the epidermis. Figures 2 and 3 and supplemental Figure 11 also show that the intracellular gradients of auxin, cytokinin, and ethylene in the epidermis are large. To the best of our knowledge, no experimental observations about this gradient have been reported.

#### Regulation of cytokinin degradation by ethylene signaling is important for predicting trends in auxin and cytokinin concentrations in mutants

Supplemental Figures 15–17 show that, in the absence of the regulation of cytokinin degradation by ethylene signaling, auxin concentration trends in mutants and patterning in auxin biosynthetic rate are predicted incorrectly (supplemental Figure 15C and 15D). Moreover, supplemental Figure 17B predicts a lower average cytokinin concentration in the *pls* mutant than in the wild type, which is opposite to experimental observations (Figure 5).

#### Regulation of cytokinin biosynthesis by auxin signaling is important for predicting auxin concentration trends in mutants and patterning of auxin biosynthetic rate

Supplemental Figures 18–20 show that, in the absence of the regulation of cytokinin biosynthesis by auxin signaling, auxin concentration trends in mutants and patterning in auxin biosynthetic rate are predicted incorrectly (supplemental Figure 18C and 18D). Moreover, supplemental Figure 20C shows that cytokinin patterning no longer emerges.

#### Role of changes in PIN1 and PIN2 concentration and patterning in various mutants

Supplemental Figures 21–24 show that changes in PIN1 and PIN2 concentration and patterning in *pin3*, *pin4*, *pin7*, or *pin3*,*4*,*7* mutants play a role in fine-tuning the concentration and patterning of auxin, cytokinin, and ethylene. Interestingly, they differentially affect different cells. For example, in the *pin3*,*4*,*7* triple mutant, if PIN1 and PIN2 are fixed at wild-type levels, the auxin concentrations in some stele and columella cells show a larger percentage increase.

#### Roles of various mutants related to ethylene signaling

In addition to the role of regulatory relationships in auxin and cytokinin concentration and patterning, modeling can also predict the roles of various mutants for future experimental validation. For example, supplemental Figures 25–28 reveal that various mutants related to ethylene signaling can show simultaneous changes in the concentration and patterning of auxin, cytokinin, and ethylene.

## Discussion

By interrogating and integrating biological knowledge, we proposed a mechanism for the simultaneous patterning of auxin and cytokinin in the *Arabidopsis* root. Based on modeling of the mechanism and experimental investigations, this research reveals that simultaneous patterning of auxin and cytokinin concentrations emerges from multi-level regulation of auxin, cytokinin, and ethylene in the root, as summarized in supplemental Figure 29.

This mechanism not only simultaneously generates auxin and cytokinin patterning that agrees with experimental observations in the *Arabidopsis* root (Figures 2 and 3) but also makes a wide range of predictions that are confirmed by experimental measurements generated by our group and independent experimental observations generated by other groups (Figures 5, 6, and 7). Therefore, we consider that the mechanism is plausible on the basis of current knowledge.

This mechanism suggests some important aspects of the simultaneous patterning of auxin and cytokinin, with a novel key role played by ethylene. The following regulatory relationships are important for simultaneous generation of auxin and cytokinin patterning. First, cytokinin promotes auxin biosynthesis (Jones et al., 2010), auxin upregulates cytokinin biosynthesis through SHY2 and IPT5 (Dello Ioio et al., 2008), and auxin promotes cytokinin biosynthesis through TM05 and LOG4 (De Rybel et al., 2014). This compares with previously proposed regulatory relationships (Moore et al., 2015) in which auxin inhibited cytokinin biosynthesis and cytokinin inhibited auxin biosynthesis (based on Nordstrom et al., 2004), which, although they generated auxin patterning similar to experimental observations, could not generate representative cytokinin patterning. Second, a novel ethylene regulatory link is also important for the simultaneous patterning of auxin and cytokinin and for prediction of a range of experimental results by the simulation. This new regulatory link is based on experimental evidence discussed in this manuscript and in Liu et al. (2017). In brief, an ETR1-dependent phospho-relay promotes ARR2 phosphorylation and activity, ARR2 promotes *CKX* expression and activity, and cytokinin degradation occurs through the action of a set of CKXs (Werner et al., 2003; 2006). These and other experimental observations (Werner et al., 2003, 2006; Hass et al., 2004; To et al., 2007; Kim et al., 2012) establish the regulation of cytokinin concentration by ethylene signaling through components of the cytokinin pathway. Inclusion of these regulatory links generates simultaneous patterning of both auxin and cytokinin similar to experimental observations and, in addition, enables the mechanism to predict a variety of experimental observations (Figures 3, 4, 5, 6, and 7).

Although the regulation of auxin, cytokinin, and ethylene concentrations in the *Arabidopsis* root is very complex (Liu et al., 2017) and the experimental data available in the literature are diverse, this research demonstrates that integration of the available experimental data can generate a plausible mechanism for studying the patterning of auxin and cytokinin as an integrative system and predicting various experimental observations. As a result, this work proposes a mechanism that enables the rational study of the complex and important simultaneous regulation of auxin and cytokinin patterning.

Because auxin and cytokinin responses are induced by their concentrations, it is reasonable to propose that auxin and cytokinin response patterning is closely related to auxin and cytokinin concentration patterning. In principle, the mechanism proposed in this work can be extended to analyze auxin and cytokinin responses by establishing links between concentration and response. However, the relationship between concentration and response is generally nonlinear. For example, modeling the response of the DII-VENUS reporter to auxin requires a model to describe the nonlinear relationship between DII-VENUS and auxin concentration (Band et al., 2012). Moreover, given the complexity of the pathways and the multiple links among pathways (Liu et al., 2017), all hormone responses will, to various degrees, be regulated by the activities of multiple hormones. Two good examples are the regulation of the cytokinin response regulator ARR5 by both ethylene and cytokinin and the regulation of the cytokinin response by both cytokinin and auxin through AHP6 (Liu et al., 2017). Thus, response modeling may require analysis of how interactions between multiple hormones determine response levels. For example, it was shown that elucidation of the cytokinin response must also take into account response regulation by auxin (De Rybel et al., 2014). A gene regulatory network involving both auxin and cytokinin establishes and maintains vascular patterning (Muraro et al., 2014). Moreover, the link from transverse auxin fluxes to lateral root initiation is regulated by both auxin and cytokinin (El-Showk et al., 2015). Importantly, all of these studies demonstrate that relationships between auxin and cytokinin concentration and response are nonlinear (Mellor et al., 2017). Thus, establishing a link between hormone concentration and response will involve carefully establishing various concentration-to-response relationships based on relevant experimental data.

Cytokinin movement in the root can be passive via diffusion but can also be regulated by cytokinin transporters. In this work, cytokinin movement was considered to occur via diffusion owing to a lack of knowledge about the distribution and kinetics of cytokinin transporters in the root (Kang et al., 2017). Once the kinetics of cytokinin transporters have been established, interesting future research can include the study of how cytokinin transporters and cytokinin diffusion work together to transport cytokinin and influence patterning and development.

Extracting a mechanism for simultaneous patterning of auxin and cytokinin necessitates simplification of the complex interactions among multiple hormones. The mechanism propose here (Figure 1) is a simplified version of the more complex network of interactions among auxin, cytokinin, and ethylene summarized in Liu et al. (2017). In this mechanism, auxin, cytokinin, and ethylene are assumed to regulate PIN1 and PIN2 in the same way. This assumption is based on analysis of a variety of experimental data (Liu et al., 2013; Moore et al., 2015; 2017). Although the main features of patterning changes in PIN1 and PIN2 concentration in *pin3*, *pin4*, and *pin7* mutants (Figure 6) can be predicted using the proposed mechanism, detection of enhanced PIN1 protein in lateral-basal membranes of the endodermis in the *pin3*, *pin4*, *pin7* triple mutant (Blilou et al., 2005) was not predicted using the mechanism. This could imply that, although the regulation of both PIN1 and PIN2 by auxin, cytokinin, and ethylene can be largely described by the same relationships as described by Figure 1, some subtle differences in their regulatory relationships may exist. Thus, the regulation of PIN1 and PIN2 may require further experimental and computational refinement in the future. Therefore, formulation of a mechanism such as Figure 1 in this work, while enabling a variety of predictions, can also identify knowledge gaps by highlighting differences between predictions and experimental observations.

With various experimental data being accumulated and new data becoming available, it is evident that elucidating the roles of the complex regulatory relationships among multiple hormones in plant development is becoming a major challenge (Schaller et al., 2015; Liu et al., 2017). This work demonstrates that integrating such data can unravel a mechanism for simultaneous patterning of auxin and cytokinin in the *Arabidopsis* root. Predictions indicate that a variety of experimental observations can also be elucidated by the same mechanism. By integrating additional hormones in the future, regulation of the concentrations and response patterning of multiple hormones in the *Arabidopsis* root can be quantitatively and rationally explored.

## Methods

### Plant materials

*Arabidopsis* seeds were obtained from lab stocks or from the Nottingham *Arabidopsis* Stock Centre (NASC). All mutant and reporter lines are in the Col-0 background, except *pls* (C24). Seedlings were grown on 10-cm square plates of half-strength Murashige and Skoog agar medium sealed with micropore tape as described previously (Chilley et al. 2006). Seedlings were grown in SANYO growth cabinets (22°C, 18-h photoperiod).

### Microscopy and image analysis

Prior to confocal imaging, TCSn:GFP, *pls-3/*TCSn:GFP, R2D2, proPIN3:PIN3:GFP, and proPIN7:PIN7:GFP seedlings were fixed using the ClearSee method previously described by Kurihara et al. (2015). The ClearSee protocol enables rapid fixing and clearing of plant tissues while retaining the activity of fluorescent proteins and is thus compatible with various fluorescent dyes (Ursache et al. 2018).

To prepare 4% paraformaldehyde (PFA) solution for the fixing procedure, 4 g of PFA powder was added to 1 l of 1× phosphate-buffered saline solution on a magnetic stirrer and heated to around 60°C. To ensure that the PFA powder was dissolved, the pH was raised using 1 M KOH until the solution was clear. The pH was then adjusted to 6.9 with 1 M HCl solution. The solution was cooled and filtered before use. The PFA solution was used fresh or kept a 4°C and used within a week.

Seedlings were transferred with forceps to the 4% PFA solution, where they were fixed under vacuum for 30 min. After fixation, seedlings were washed in 1× phosphate-buffered saline solution twice before the addition of ClearSee solution, in which they were again placed under vacuum for 30 min.

ClearSee solution was prepared via mixing xylitol (10% w/v), sodium deoxycholate (15% w/v), urea (25% w/v), and H_2_O in solution for 30 min. Fixed seedlings were left in ClearSee solution at room temperature for at least one week, and the ClearSee solution was replaced every few days. After clearing, seedlings were stained and imaged.

Seedlings were examined using a Zeiss LSM 800 or LSM 880 laser-scanning confocal microscope (LSCM). Roots were imaged using either a 10× or 20× air objective lens. z stacks were obtained for each seedling to gain as much information as possible. Settings such as gain, line, z step, averaging, etc., were altered between each fluorescent reporter to optimize image quality and consistency.

To visualize cell structure and organization under the LSCM, cleared seedlings were submerged in 0.1% Calcoflour White in ClearSee solution for 30 min. After staining, Calcolfuor White solution was replaced by ClearSee, and seedlings were washed for another 30 min. For LSCM observation, fixed seedlings were mounted on slides in ClearSee solution under a coverslip.

### Digital root construction and spatiotemporal modeling

The details for construction of a digital root, numerical methods, averaging hormone concentration for a cell, and averaging hormone concentration in the digital root have been reported previously (Moore et al., 2015; 2017; Liu et al., 2017) and remain the same for this research. In particular, digital root structure with actual cell geometries, polar localization of efflux carriers, and nonpolar localization of influx carriers have been described in detail (Liu et al., 2017; Moore et al., 2017). The images of PIN1,2,3,4,7 and AUX1 and LAX2,3 were shown in Moore et al., (2017). The parameter values for modeling equations in this research are included in supplemental Tables 1–3.

### Comparison of modeling and experimental results

Comparison of modeling and experimental results focused on longitudinal trends along the root; we did not attempt to make quantitative comparison at the cell or pixel level for several reasons. (1) Although the modeled digital root was constructed on the basis of typical *Arabidopsis root* anatomy and included important root features such as the type, geometry, size, and wall of each cell and the extracellular matrix (Moore et al., 2015; Liu et al., 2017), the size, geometry, and wall locations of cells in the digital root were not exactly the same as those of their counterparts in experimental images of individual roots. Therefore, it is impossible to make direct quantitative comparisons with experimental images at the cell or pixel level. (2) Experimental images in our experiments and those in the literature generally show trends and patterning of the measured components and do not quantify actual concentrations of any components. Thus, direct concentration comparison is less important than trend and pattern comparison. (3) Biologically, patterning generally refers to trend or gradient change and is considered to play a crucial role in root development. Thus, comparisons between modeled and experimental results in this research concentrate on similarities or differences in trends and patterning.

## Funding

J.L. and K.L. gratefully acknowledge 10.13039/501100000690Research Councils UK and the 10.13039/501100000268Biotechnology and Biological Sciences Research Council (BB/E006531/1) for funding in support of this study. G.J. acknowledges receipt of a BBSRC DTP studentship (BB/M011186/1). C.C. gratefully acknowledges the Advanced Foreign Experts Project (G2023157014L) and the Cultivating Fund Project of Hubei Hongshan Laboratory (2022hspy002).

## Author contributions

J.L. and K.L. initiated the project. S.M., J.L., and K.L. designed the modeling and the experimental study and drafted the manuscript. J.L., K.L., J.F.T., and C.C. supervised the study. S.M., J.L., and G.J. carried out modeling and experimental work. All authors edited the final draft of the manuscript.

## Supplemental information

Document S1. Supplemental Figures 1–29 and supplemental Tables 1–3

Document S2. Article plus supplemental information
