# Supplementary material for: A predictive model for ethylene-mediated auxin and cytokinin patterning in the Arabidopsis root
Source: Plant Commun. 2024 Mar 19;5(7):100886. doi: 10.1016/j.xplc.2024.100886 (PMC11287175; doi:10.1016/j.xplc.2024.100886)
Supplement: Document S1. Supplemental Figures 1–29 and supplemental Tables 1–3 [file mmc1.pdf]

Plant Communications, Volume *5*

Supplemental information

A predictive model for ethylene-mediated auxin and cytokinin patterning in the *Arabidopsis* root

Simon Moore, George Jervis, Jennifer F. Topping, Chunli Chen, Junli Liu, and Keith Lindsey

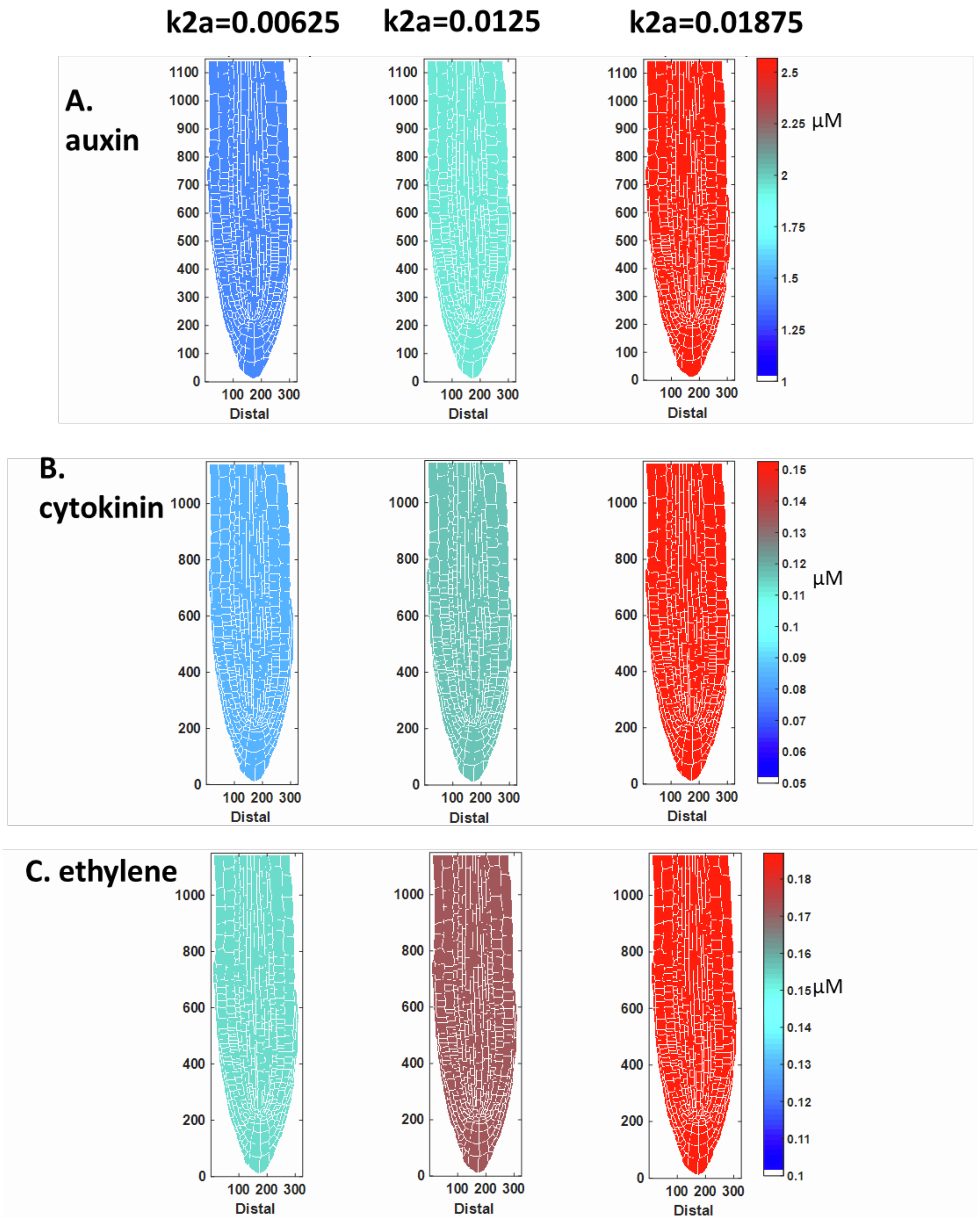

Figure S1. An example of simultaneously enhancing the concentration of (A) auxin, (B) cytokinin and (C) ethylene by increasing auxin biosynthesis rate in homogenous cells of the root.

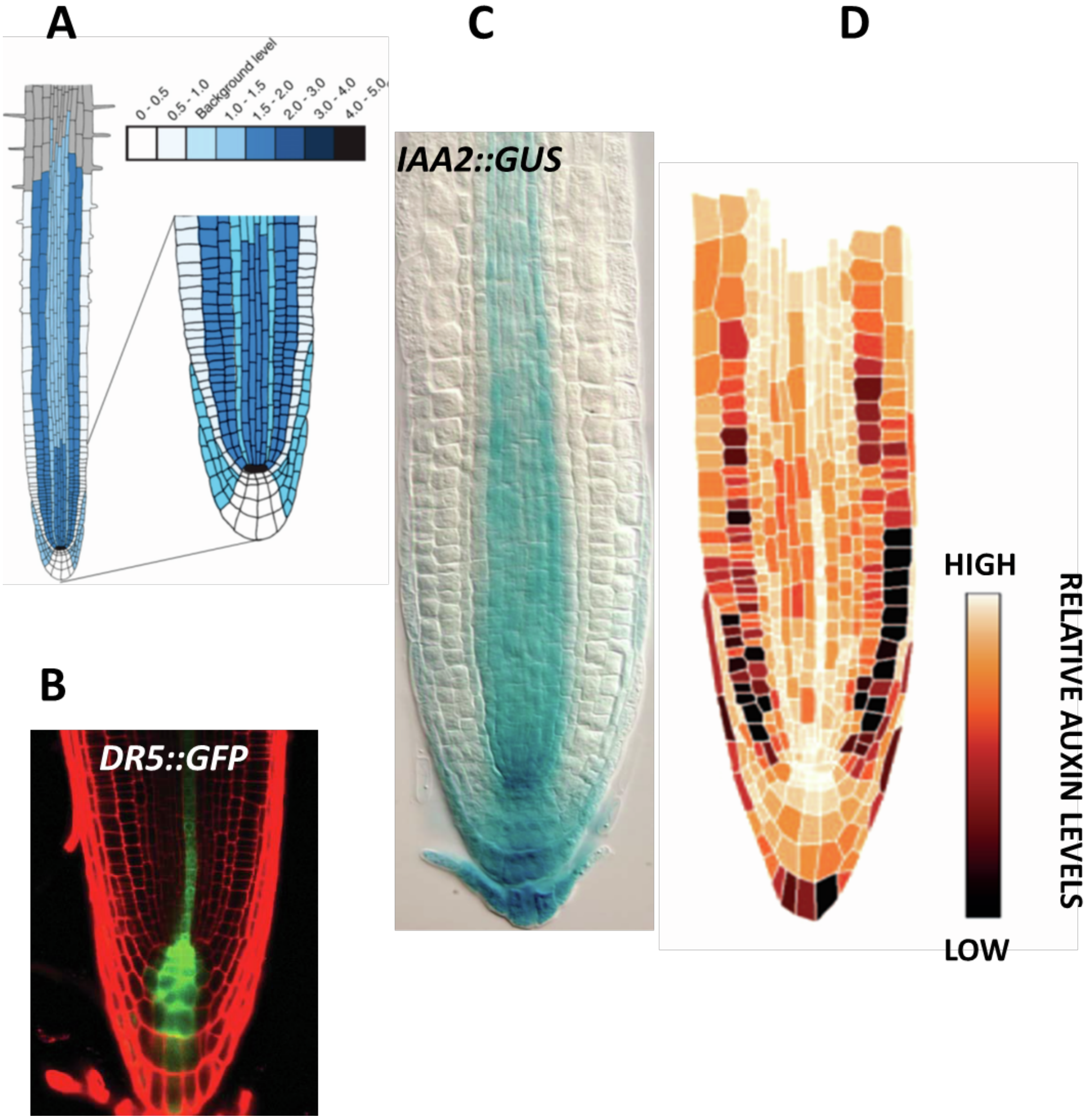

Figure S2. Experimental observations for auxin: including IAA distribution (A; Figure 3 in Petersson et al., 2009), auxin response patterning *DR5::GFP* and *IAA2::GUS* (B, C; Figures 3 and 4 in Grieneisen et al., 2007), and relative auxin levels based on the DII-VENUS inverse auxin response reporter (D; Figure 2 in Brunoud et al., 2012).

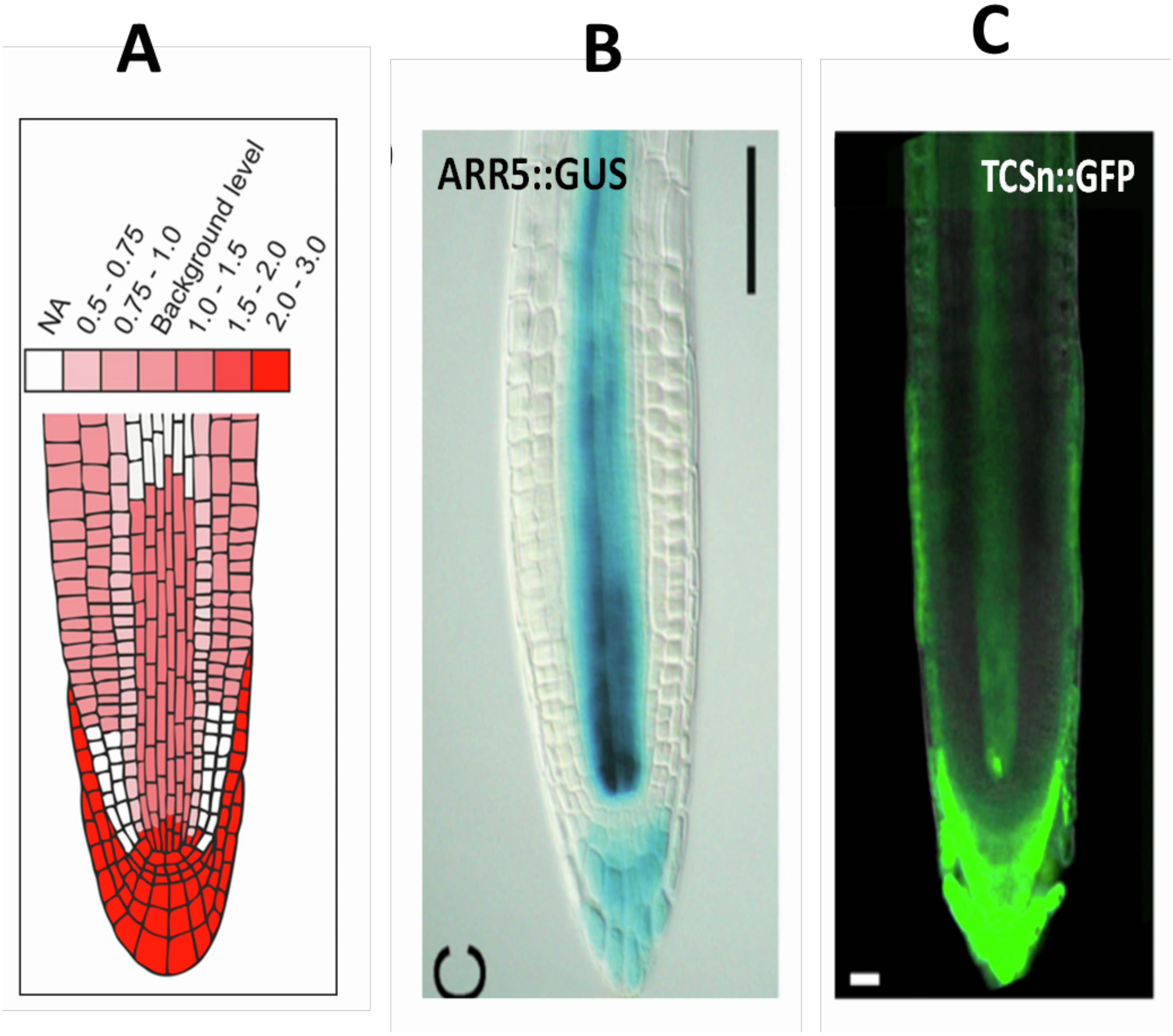

Figure S3. Experimental observations about cytokinin. The relative cytokinin concentration measured using cell sorting and mass spectrometry (A; Figure 5 in Antoniadi et al., 2015) indicate high cytokinin concentrations in the QC region, columella and lateral root cap, medium concentrations in the vascular cylinder and in the epidermis and cortex in the TZ and EZ, and lower concentrations in the endodermis. The in silico concentration patterning (Figures 3A, 3C to 3F) is very similar to these experimental observations. Cytokinin response was also measured using the ARR5::GUS reporter (B; Figure 3 in Werner et al., 2003) or using the TCSn::GFP reporter (C; Figure 4 in Zurcher et al., 2013).

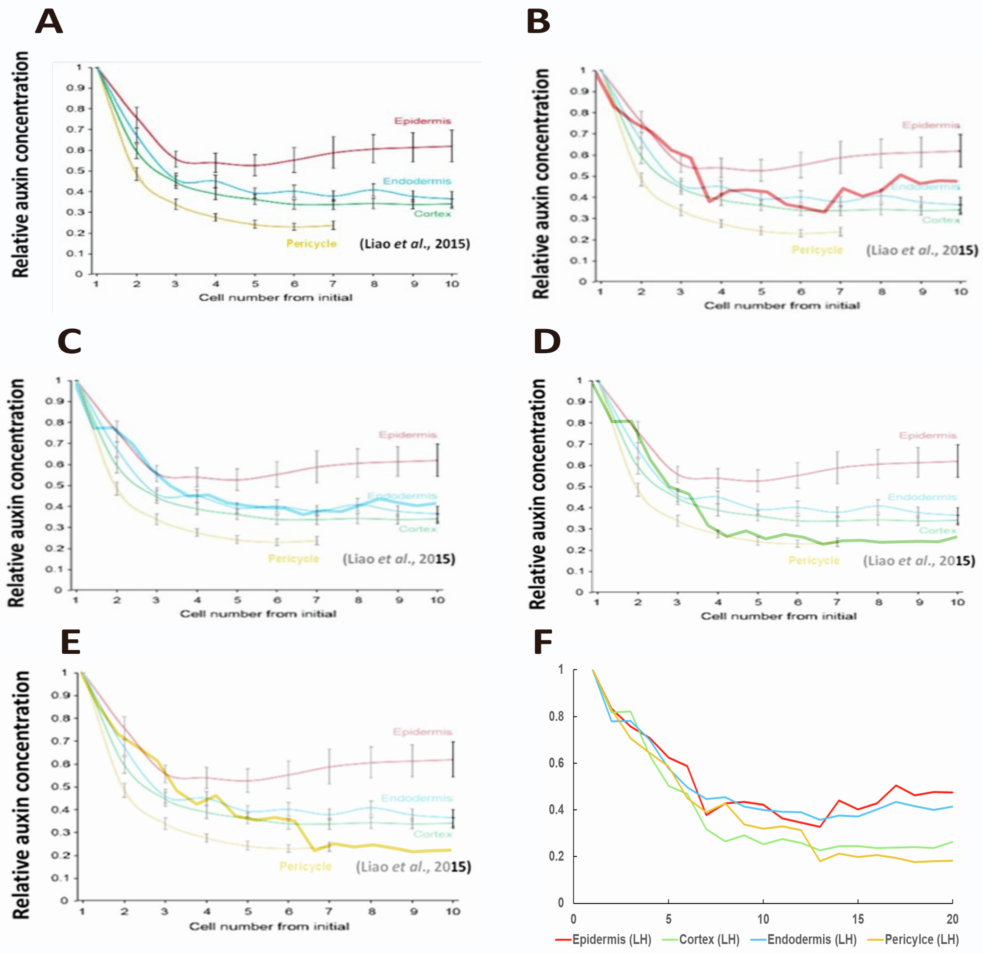

Figure S4. A. Experimental observations of auxin response trends in cell files above the initials. Relative auxin concentration trends in the epidermis, cortex, endodermis and pericycle cells files above the initials were experimentally established using the R2D2 reporter (Supplementary Figure 6 in Liao et al. 2015). B, C, D and E: Superimposing modelled curve in Figure 5A in the main text for the epidermal, cortical, endodermal and pericycle cells (top four curves in Figure 5 in the main text) over experimental results (Figure S4A). in B, two red-coloured curves are compared. In C, two blue-coloured curves are compared. In D, two light-green-coloured curves are compared. In E, two yellow-coloured curves are compared. For the superimposition, the following is noted: the cell size, geometry or wall location in the digital root is not the same as its counterpart in experimental image. To make the comparison, the "1" point at y-axis for the modelled curve is approximately aligned with the "1" point at y-axis of experimental curves (Figures S4A; Supplementary Figure 6 in Liao et al. 2015). The y-axis in both modelled and experimental curves represent relative auxin concentration. In F, the modelled four curves for four cell files in top four panels of Figure 5A in the main text are displayed together for comparison.

A

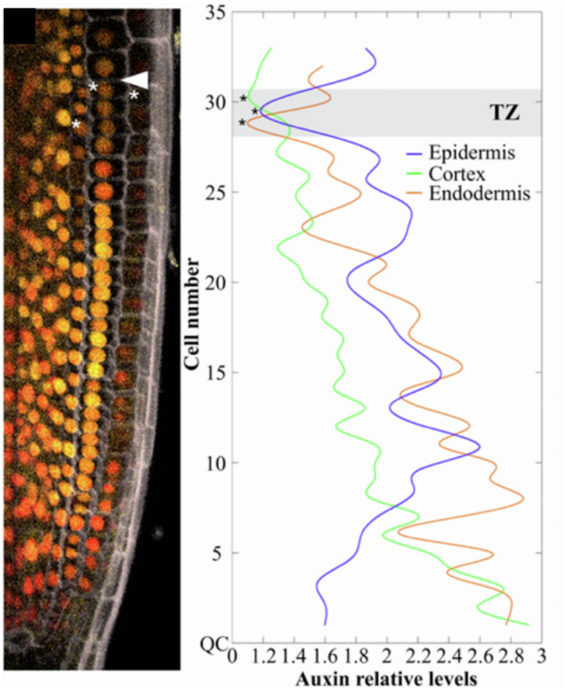

B

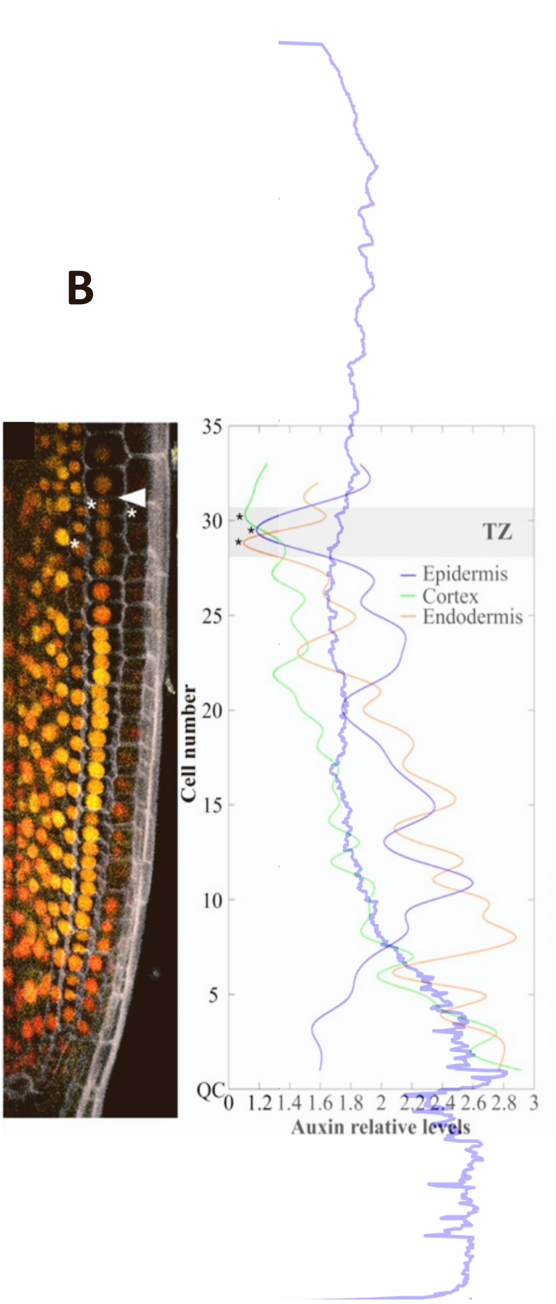

Figure S5. A. Experimental observations of auxin response minimum in the transition zone. Experimental results indicate that a minimum in the auxin response along the root axis (Figure 3 in Di Mambro et al. 2017) occurs at the boundary of the proximal meristem and distal transition zone. B. Superimposing modelled curve in Figure 5B in the main text over experimental results (Figure S5A). The transition zone in Figure 5B in the main text is located approximately between 650 μm and 750 μm. To make the comparison, the middle of transition zone in the modelled curve of Figure 5B in the main text is aligned with the middle of the grey zone of experimental curves (Figure S5A). For the superimposition, the following is noted: the cell size, geometry or wall location in the digital root is not the same as its counterpart in experimental image.

A

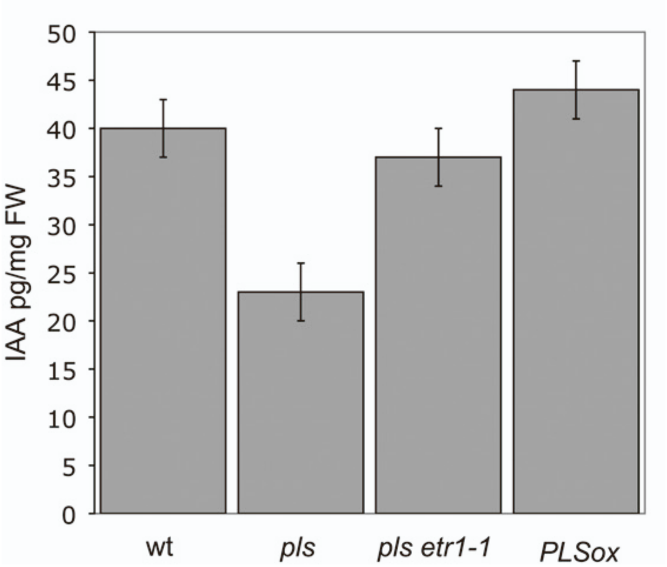

B

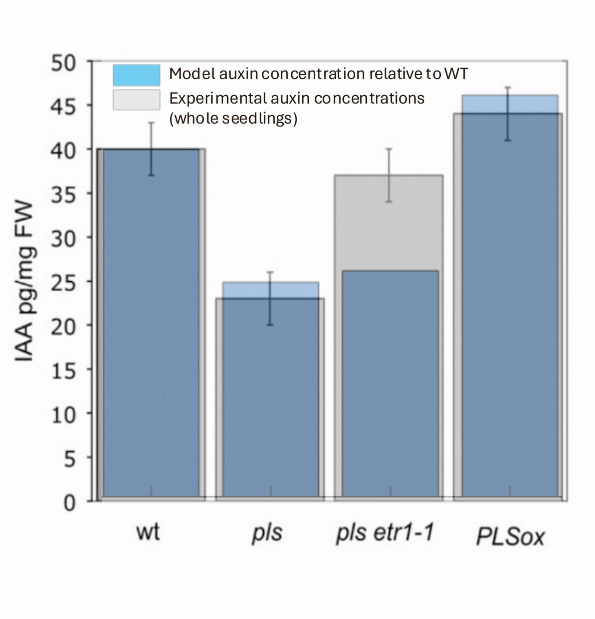

Figure S6. A. Experimental observations of average auxin concentration trends in WT, *pls* mutant, *pls etr1* double mutant, and PLSox (Figure 4C in Chilley et al. 2006). B. Superimposing modelled results in Figure 5C in the main text over experimental results (Figure S6A). It is noted that experimental results were obtained using whole seedling while modelling results are root only using the digital root.

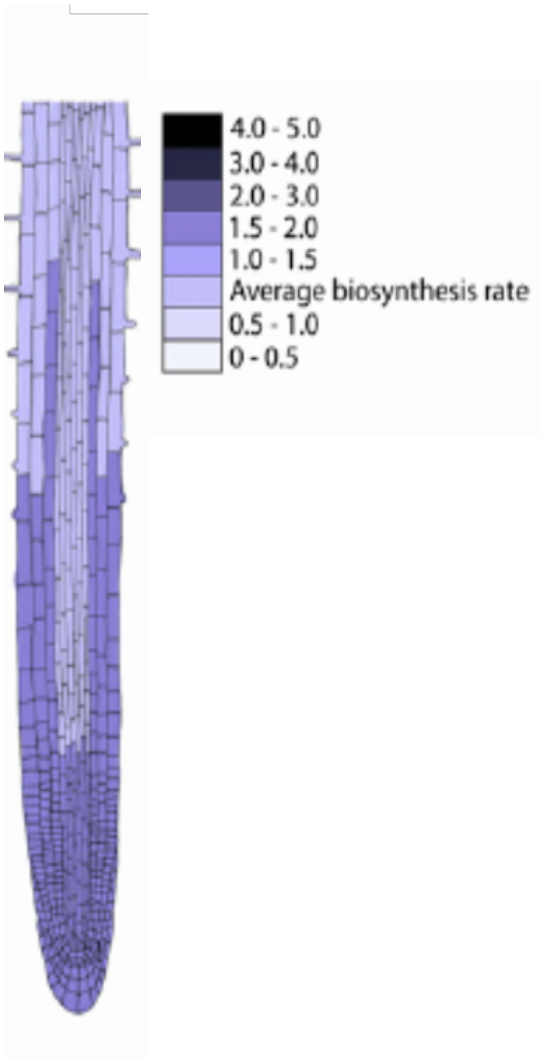

Figure S7. Experimental observations of patterning the rate of auxin biosynthesis (Figure 5 in Petersson et al. 2009).

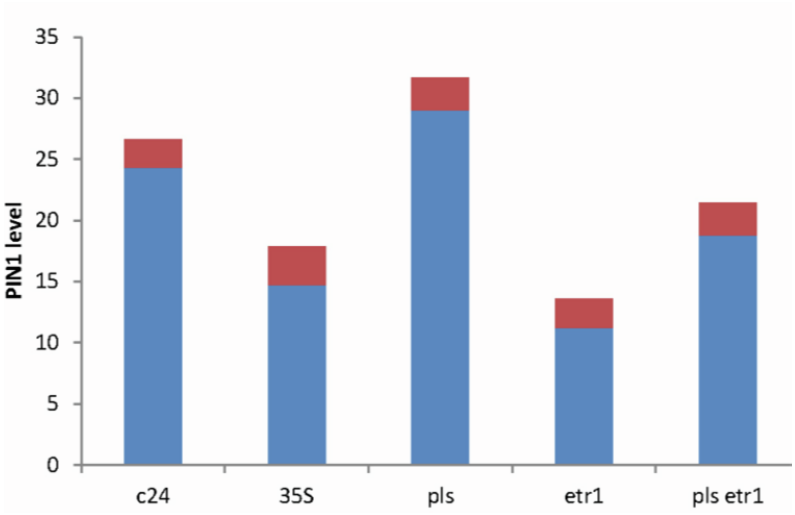

Figure S8. Experimental observations of the average concentration trend of PIN1 and PIN2 proteins in WT, PLSox transgenic, *pls* and *etr1* mutants, and *pls etr1* double mutant (Figure 1B in Liu et al. 2013).

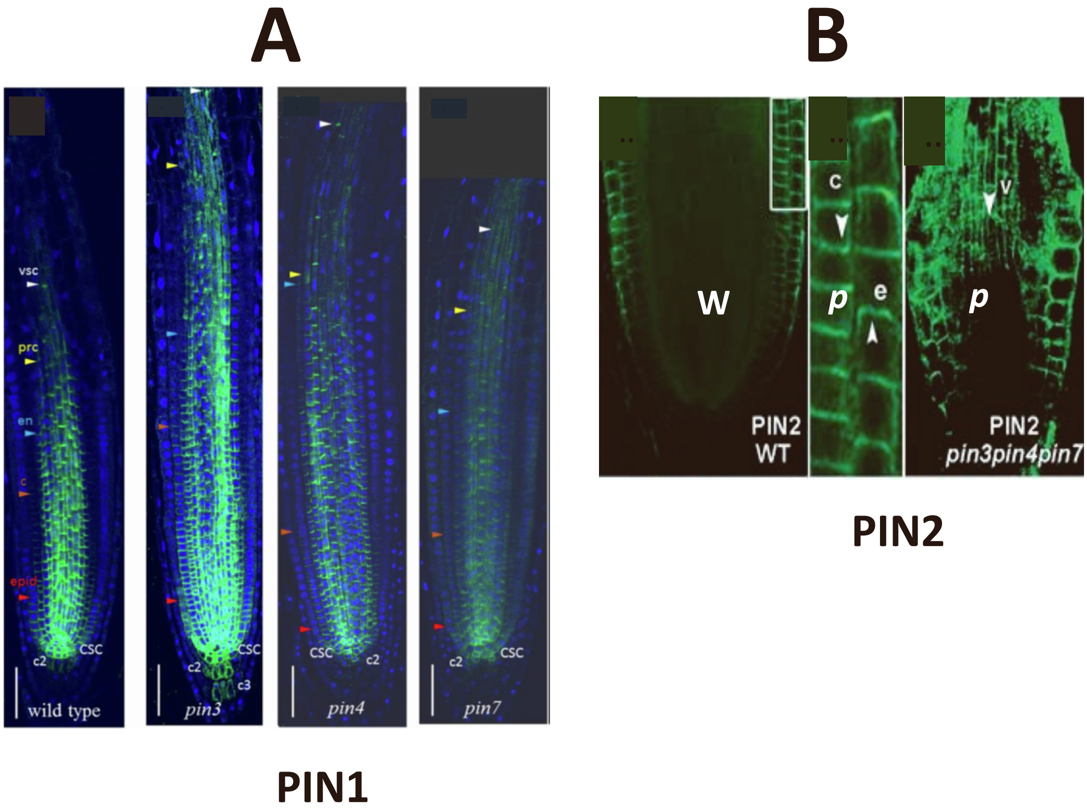

Figure S9. Experimental observations of changes in PIN1 and PIN2 concentrations in *pin3*, *pin4*, *pin7* mutants. A. The region of PIN1 expression extends shootward up the vasculature in these mutants (Figure S9A; Figure 6 in Omelyanchuk et al. 2016). B. A clear increase in PIN2 level in the vasculature emerges (Figure S9B; Figure 1 in Blilou et al. 2005).

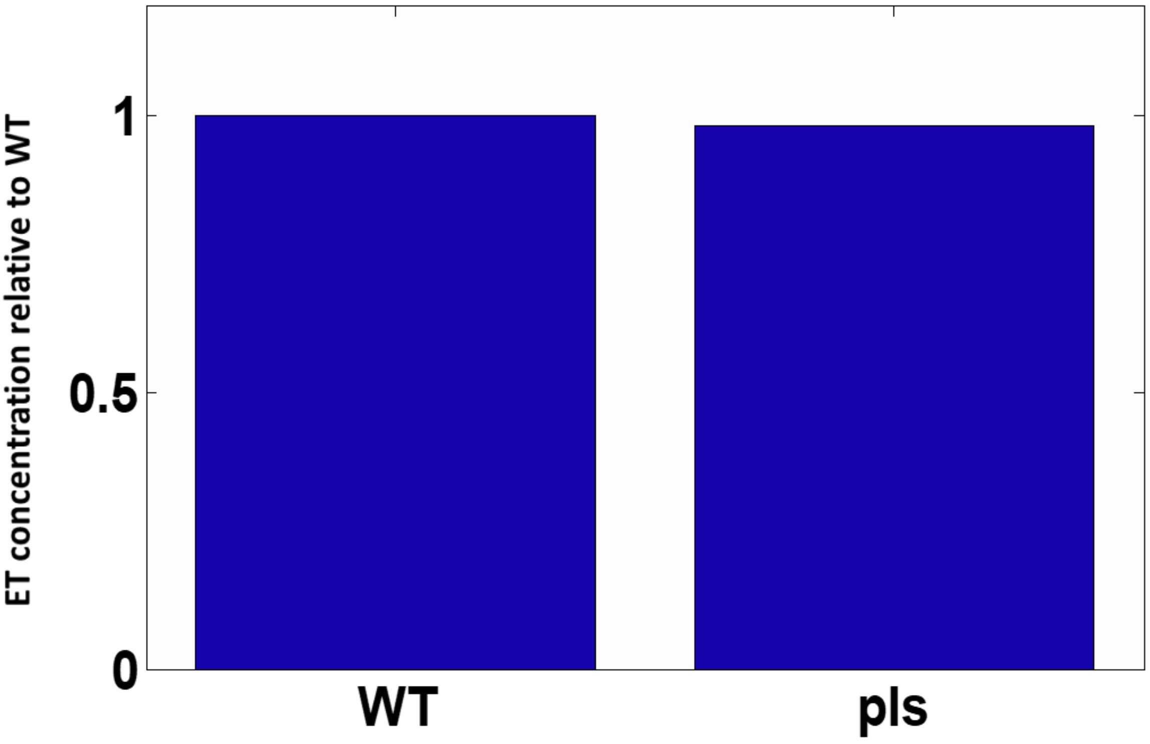

Figure S10. Modelled ethylene concentration in wild type (WT) and the *pls* mutant. This is consistent with experimental observations (Chilley et al., 2006).

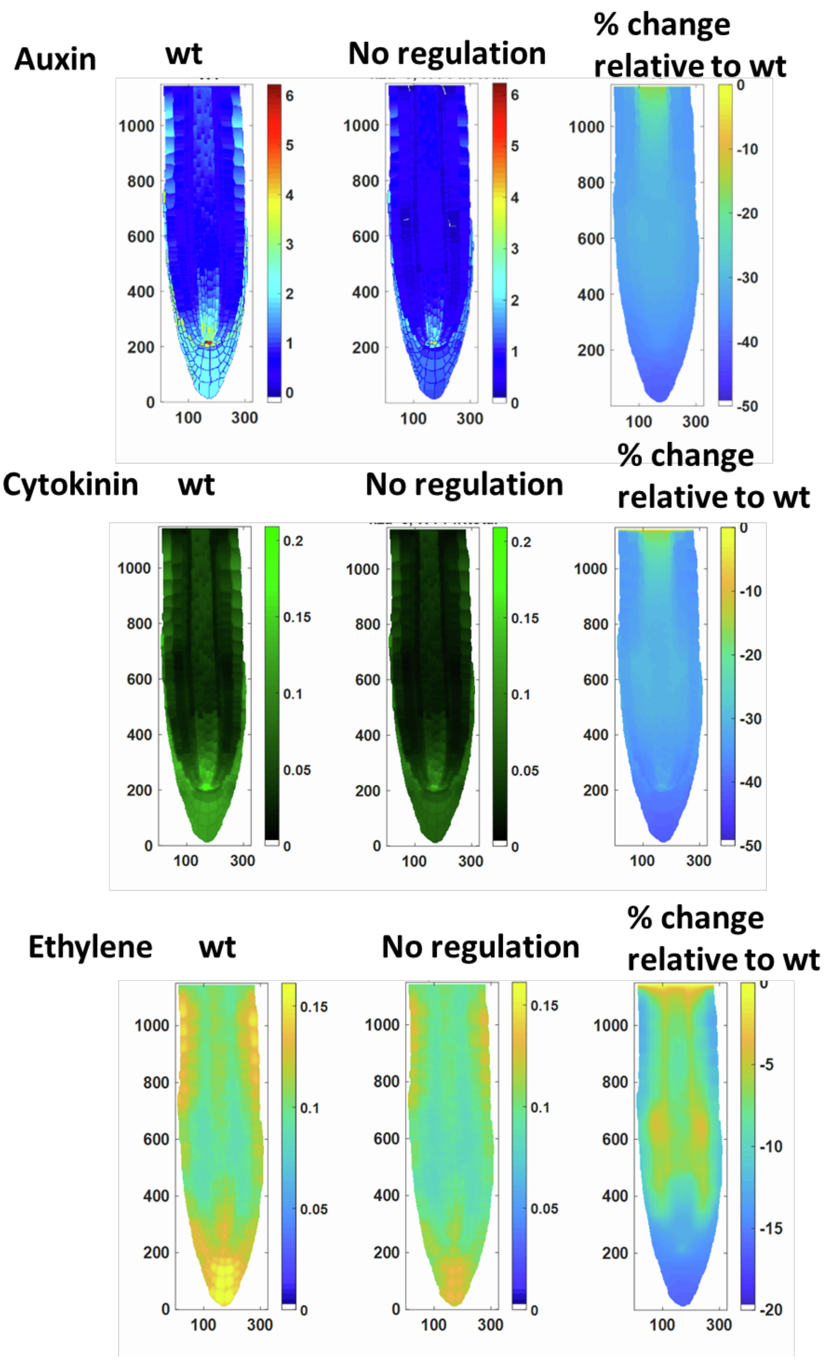

.

Figure S11. Modelling reveals that auxin patterning still emerges when auxin biosynthesis is not regulated by cytokinin or ethylene (k2a=0) with all auxin transporters being fixed as those in the wildtype. Moreover, both cytokinin and ethylene patterning still emerges.

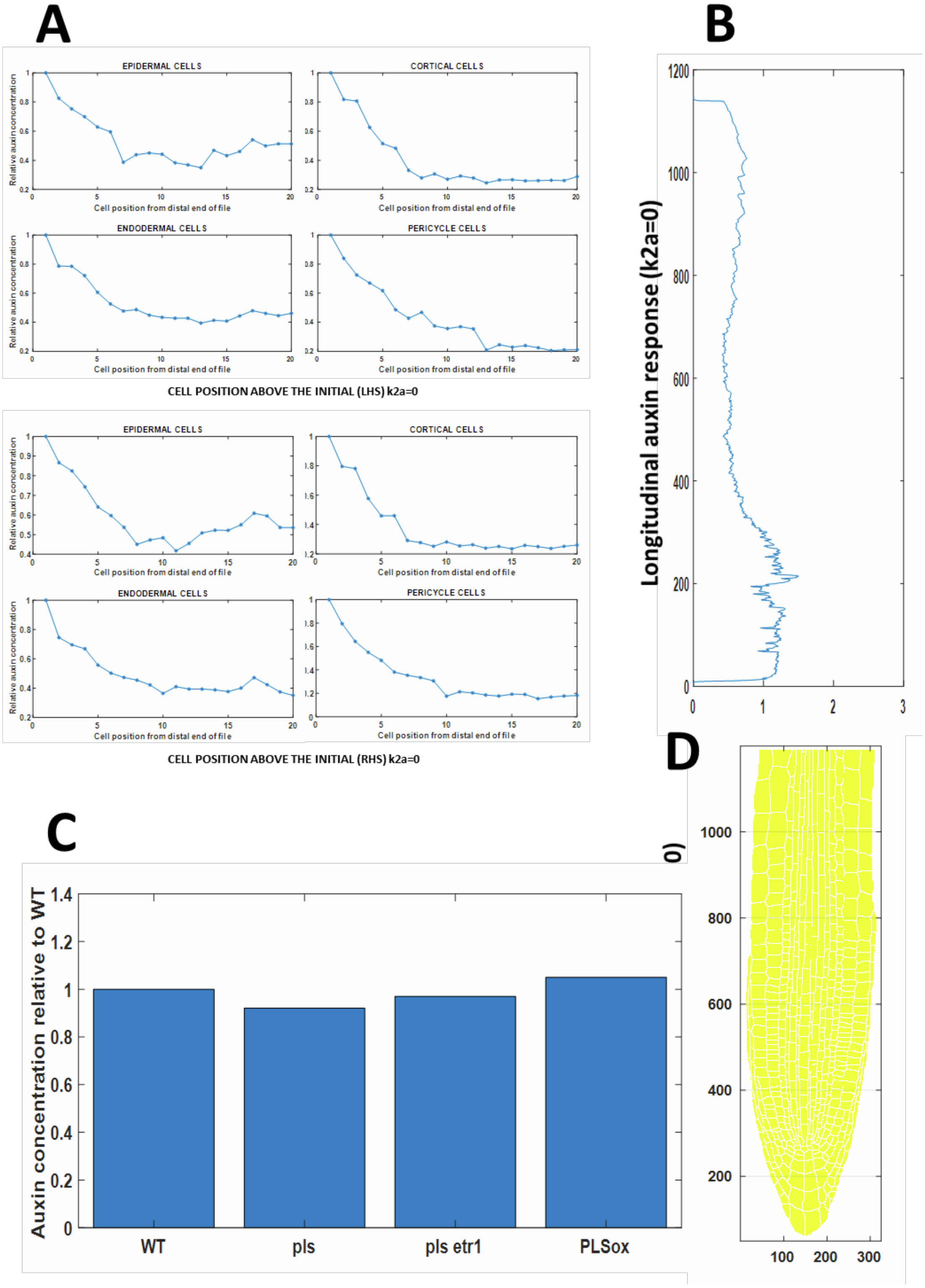

Figure S12. Same as Figure 5 in the main text, but in the absence of auxin biosynthesis regulation (k2a=0).

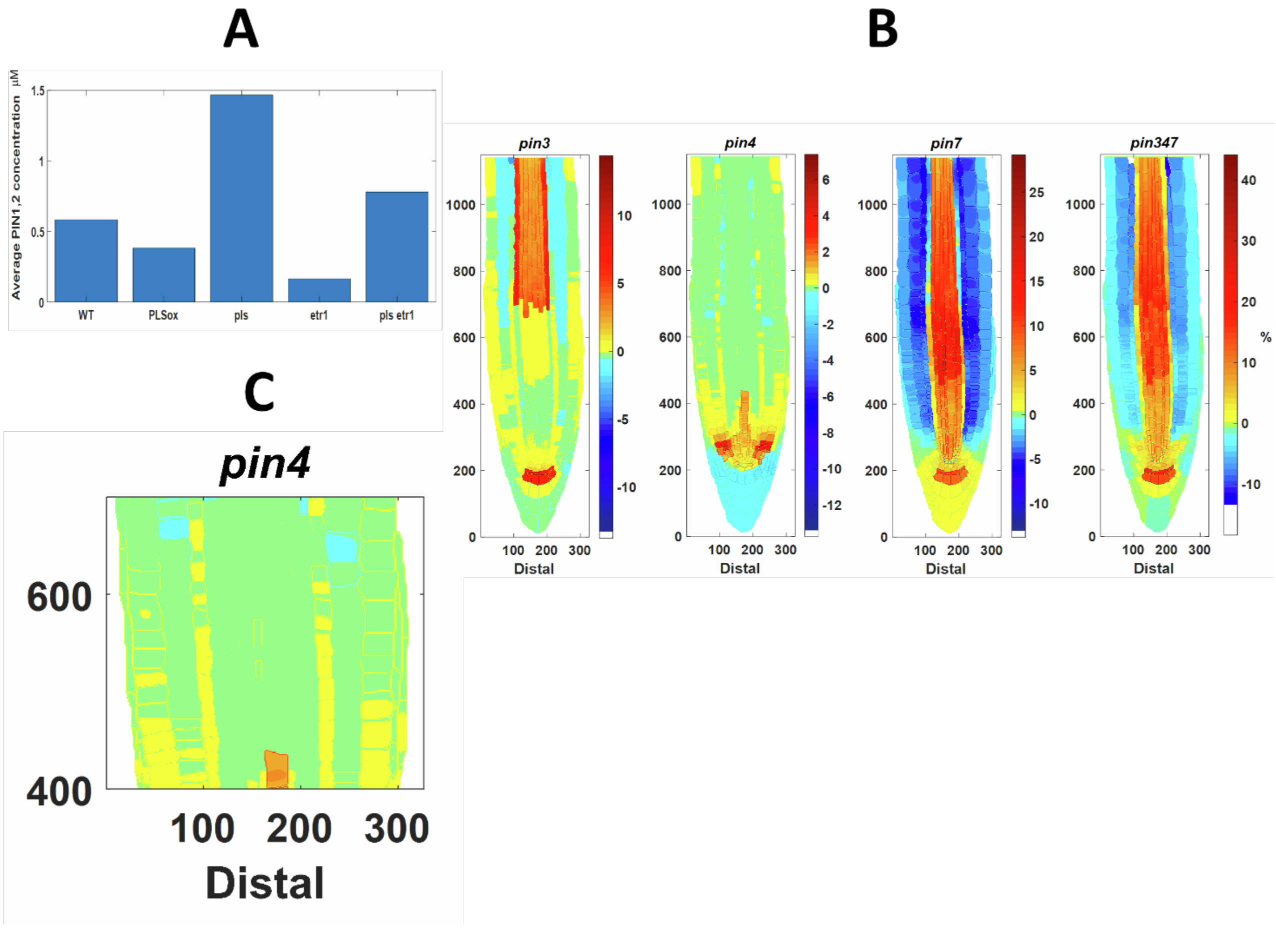

Figure S13. Same as Figure 6 in the main text, but in the absence of auxin biosynthesis regulation (k2a=0).

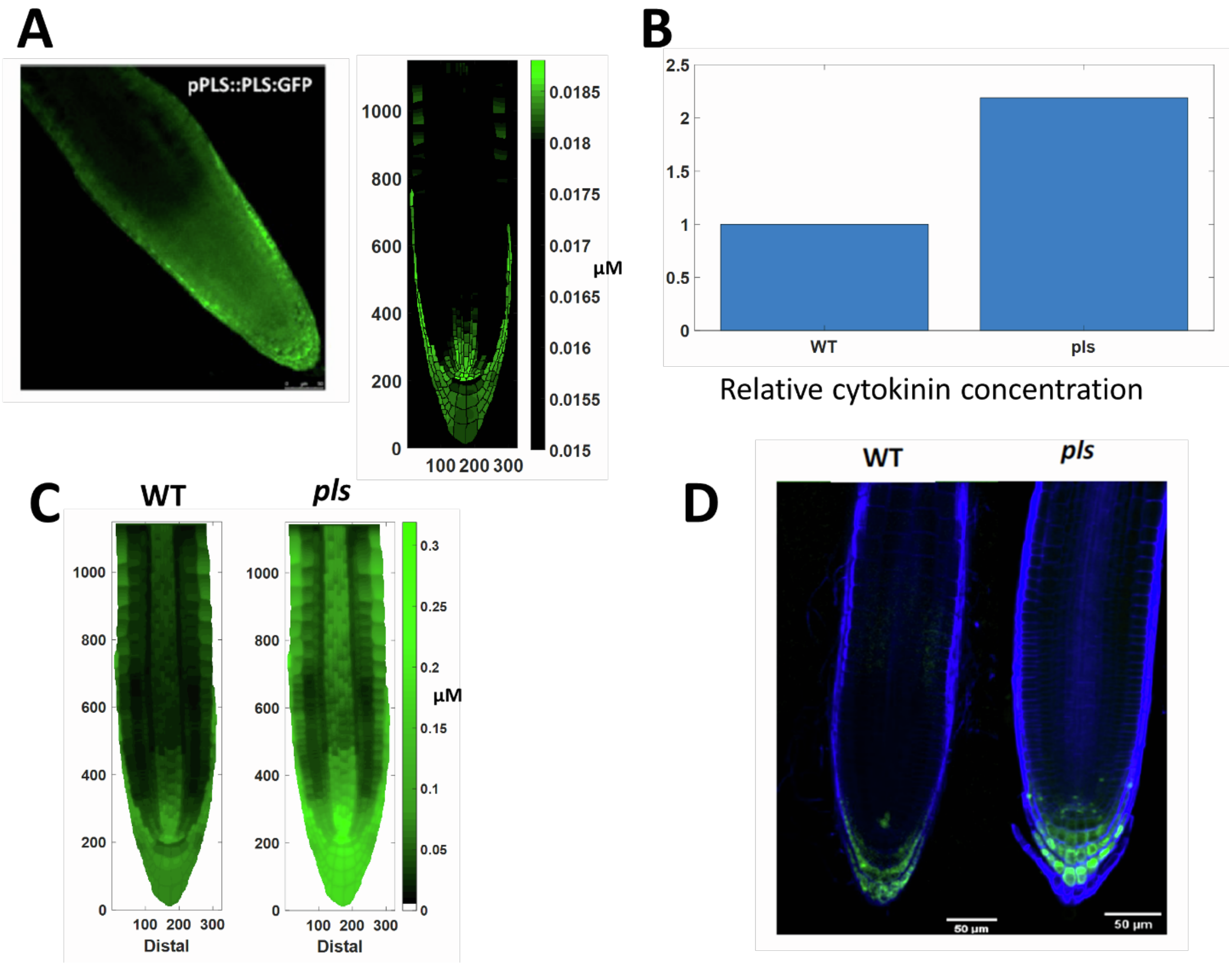

Figure S14. Same as Figure 7 in the main text, but in the absence of auxin biosynthesis regulation (k2a=0).

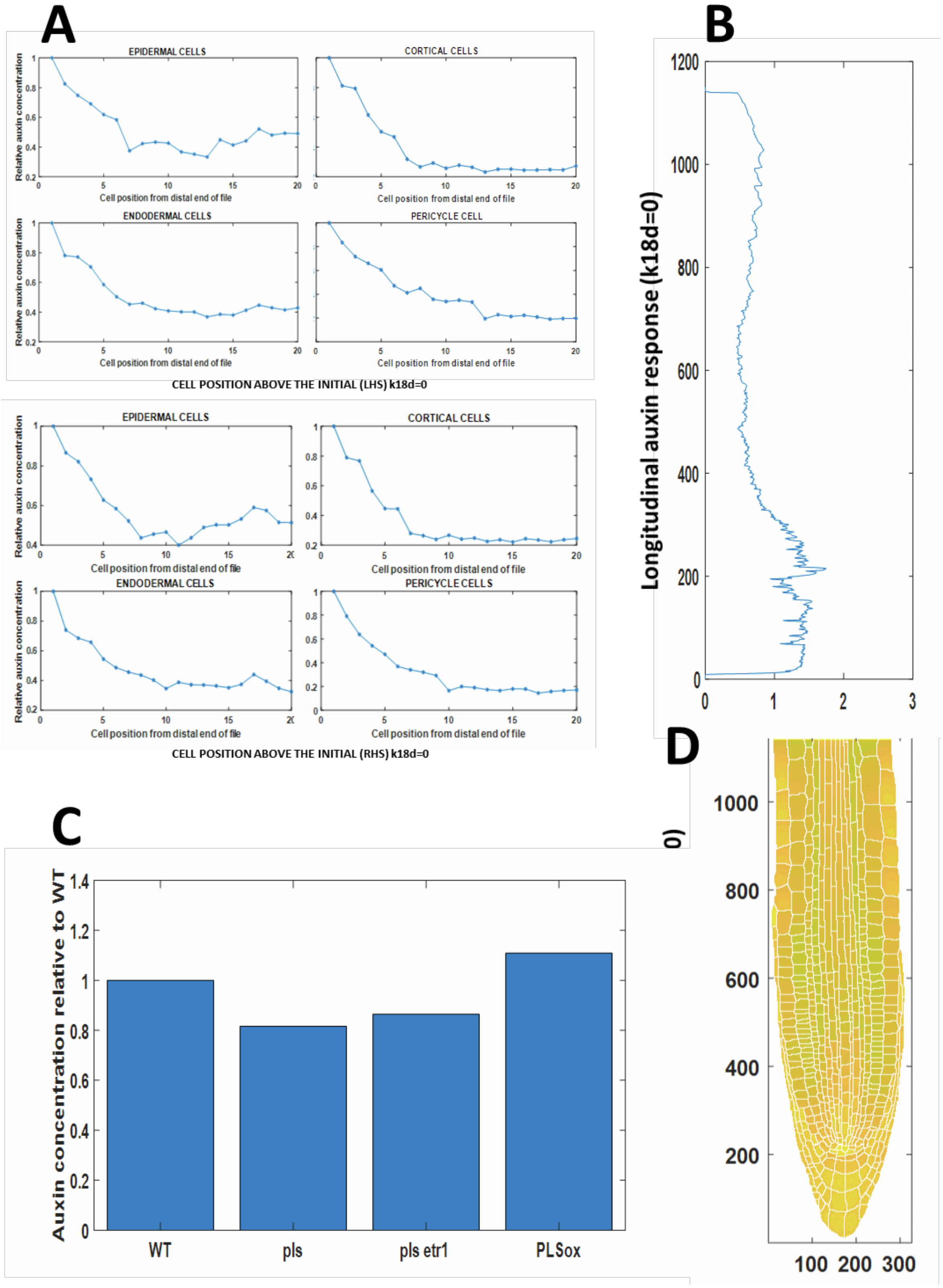

Figure S15. Same as Figure 5 in the main text, but in the absence of the regulation of cytokinin degradation by ethylene signalling (k19a=0).

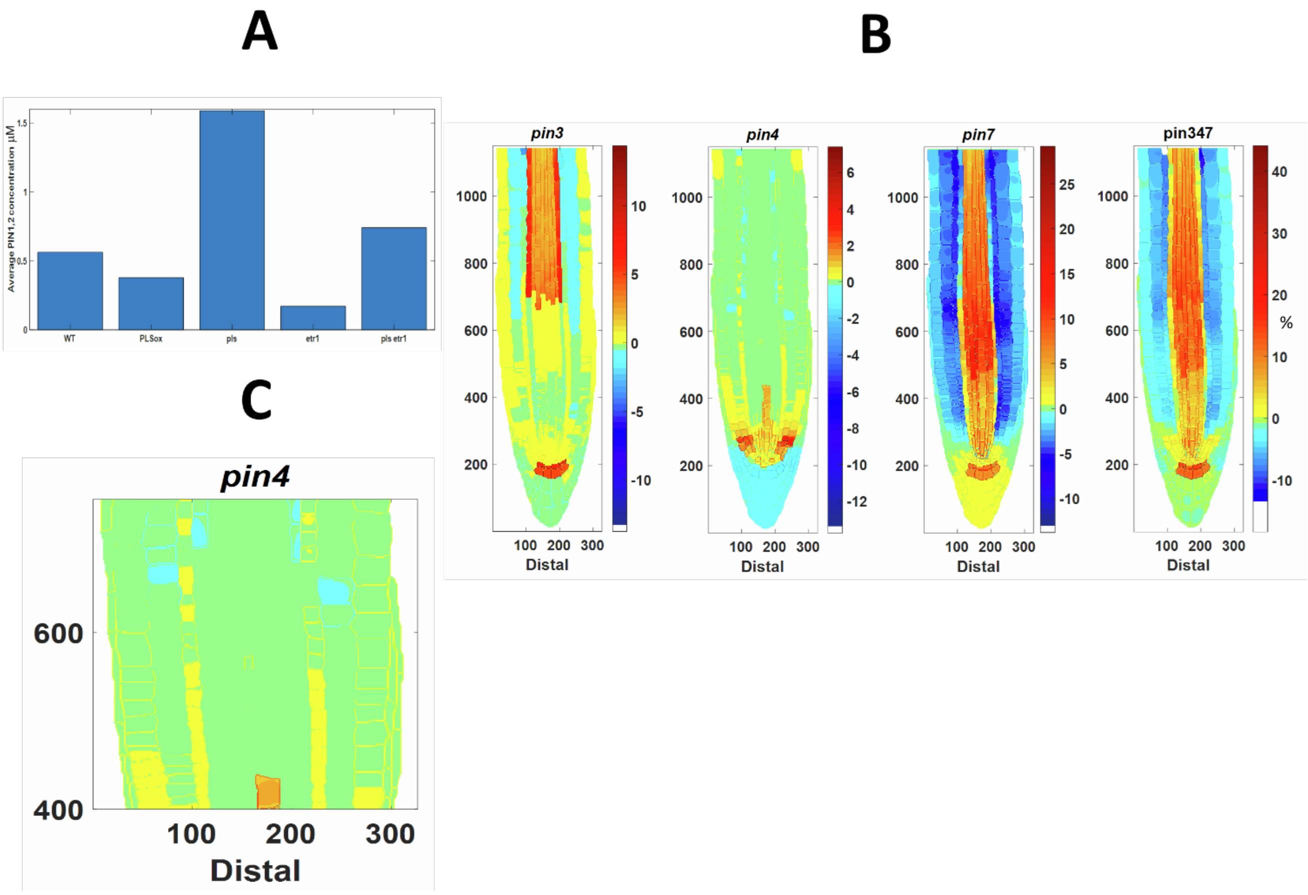

Figure S16. Same as Figure 6 in the main text, but in the absence of the regulation of cytokinin degradation by ethylene signalling (k19a=0).

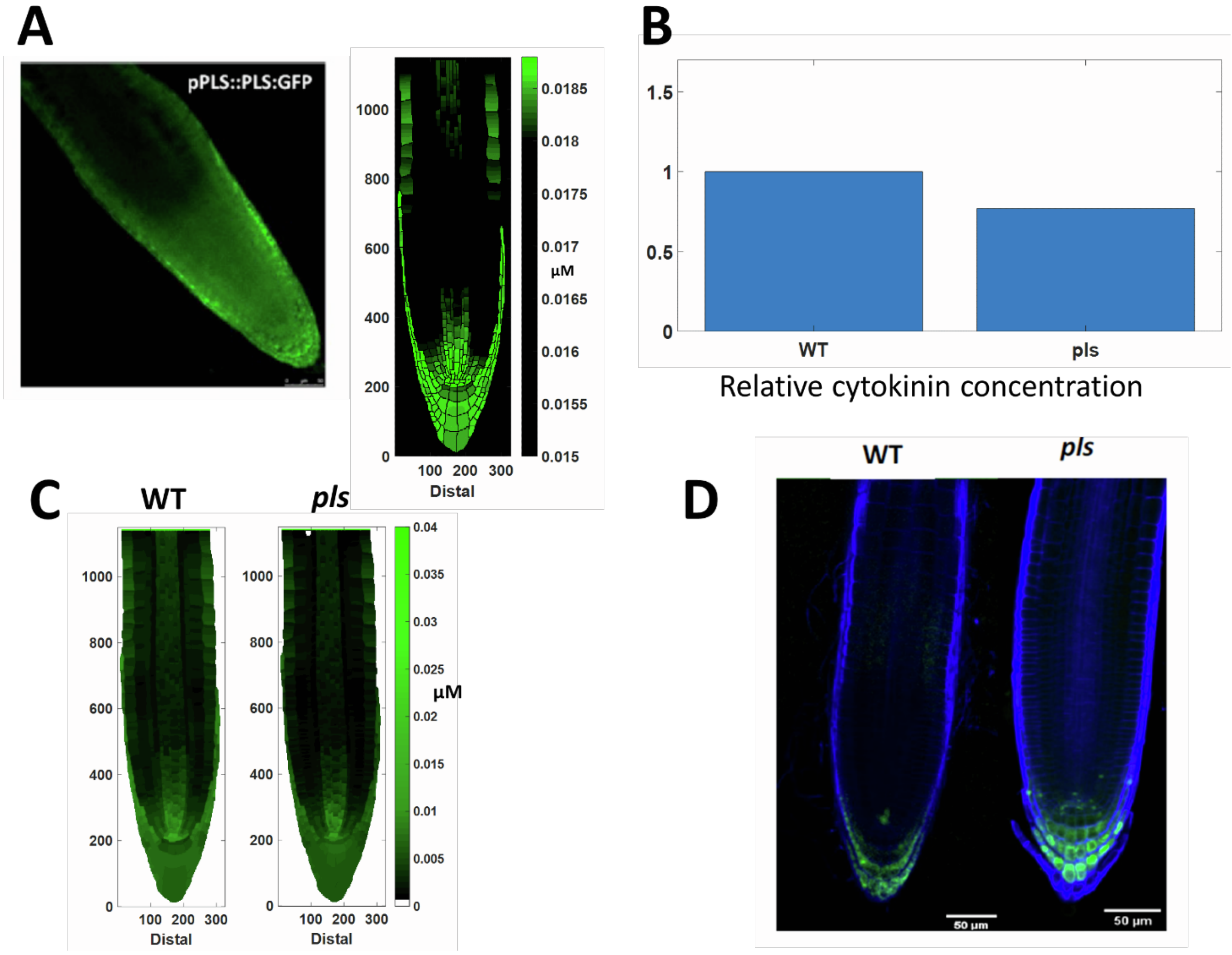

Figure S17. Same as Figure 7 in the main text, but in the absence of the regulation of cytokinin degradation by ethylene signalling (k19a=0).

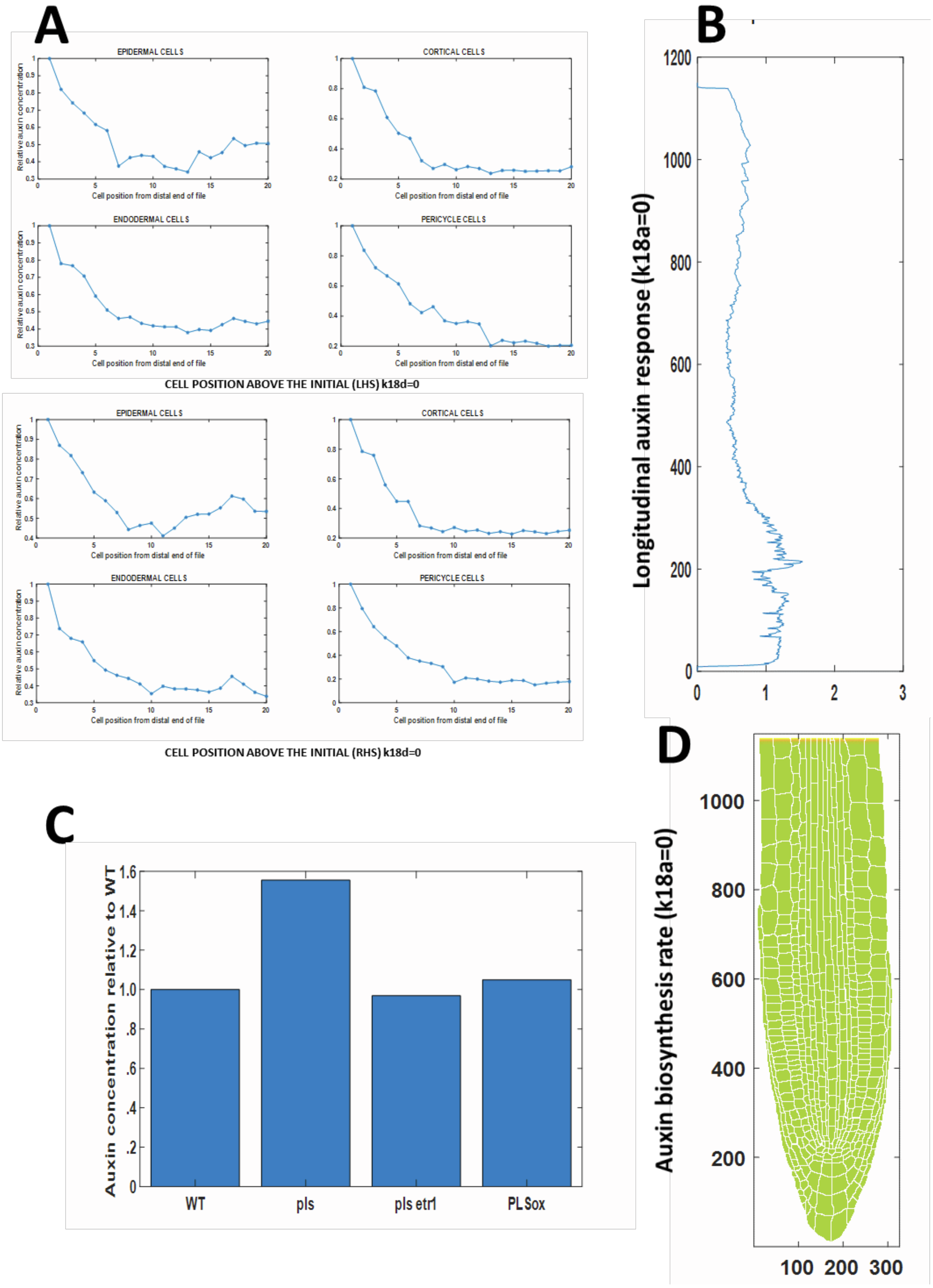

Figure S18. Same as Figure 5 in the main text, but in the absence of the regulation of cytokinin biosynthesis by auxin signalling (k18a=0).

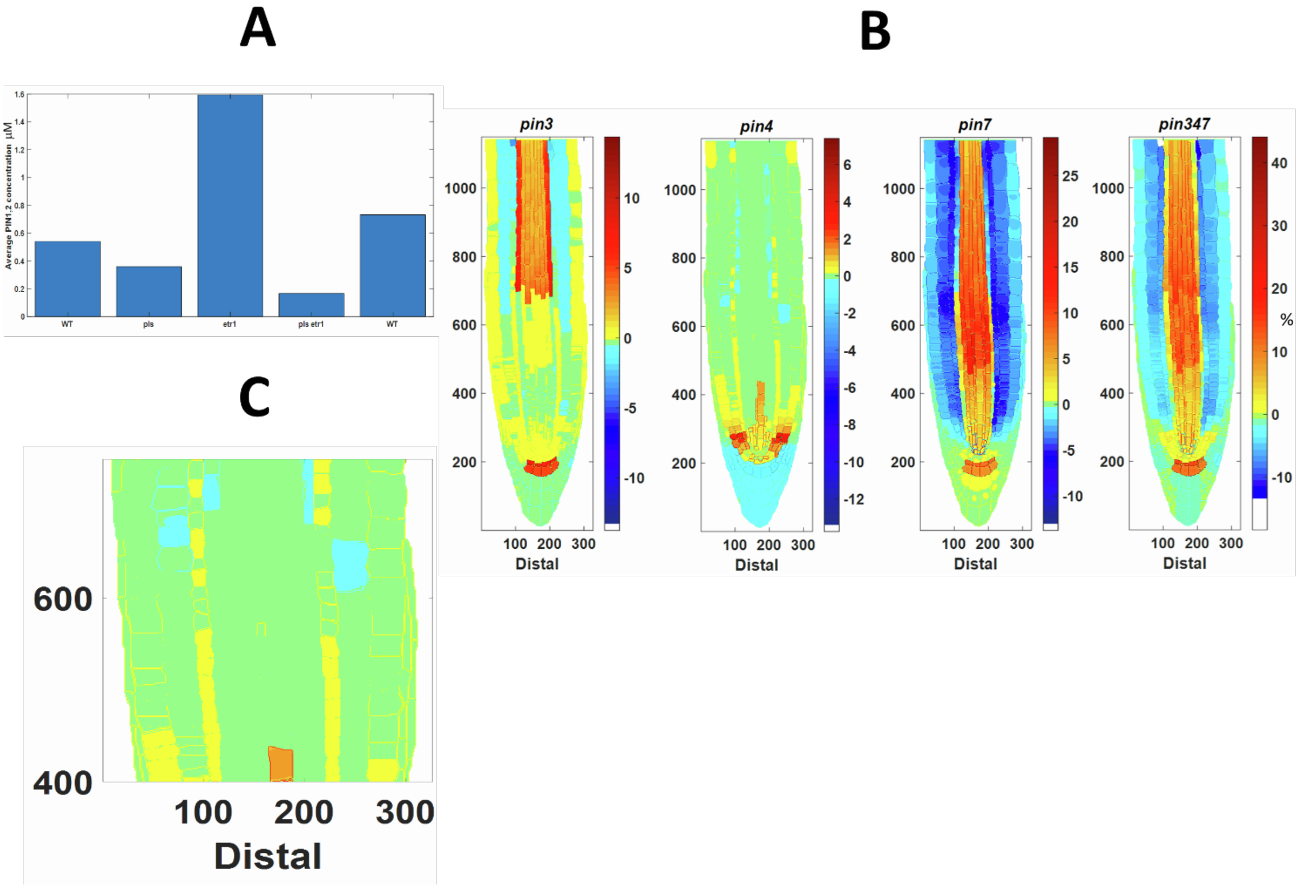

Figure S19. Same as Figure 6 in the main text, but in the absence of the regulation of cytokinin biosynthesis by auxin signalling (k18a=0).

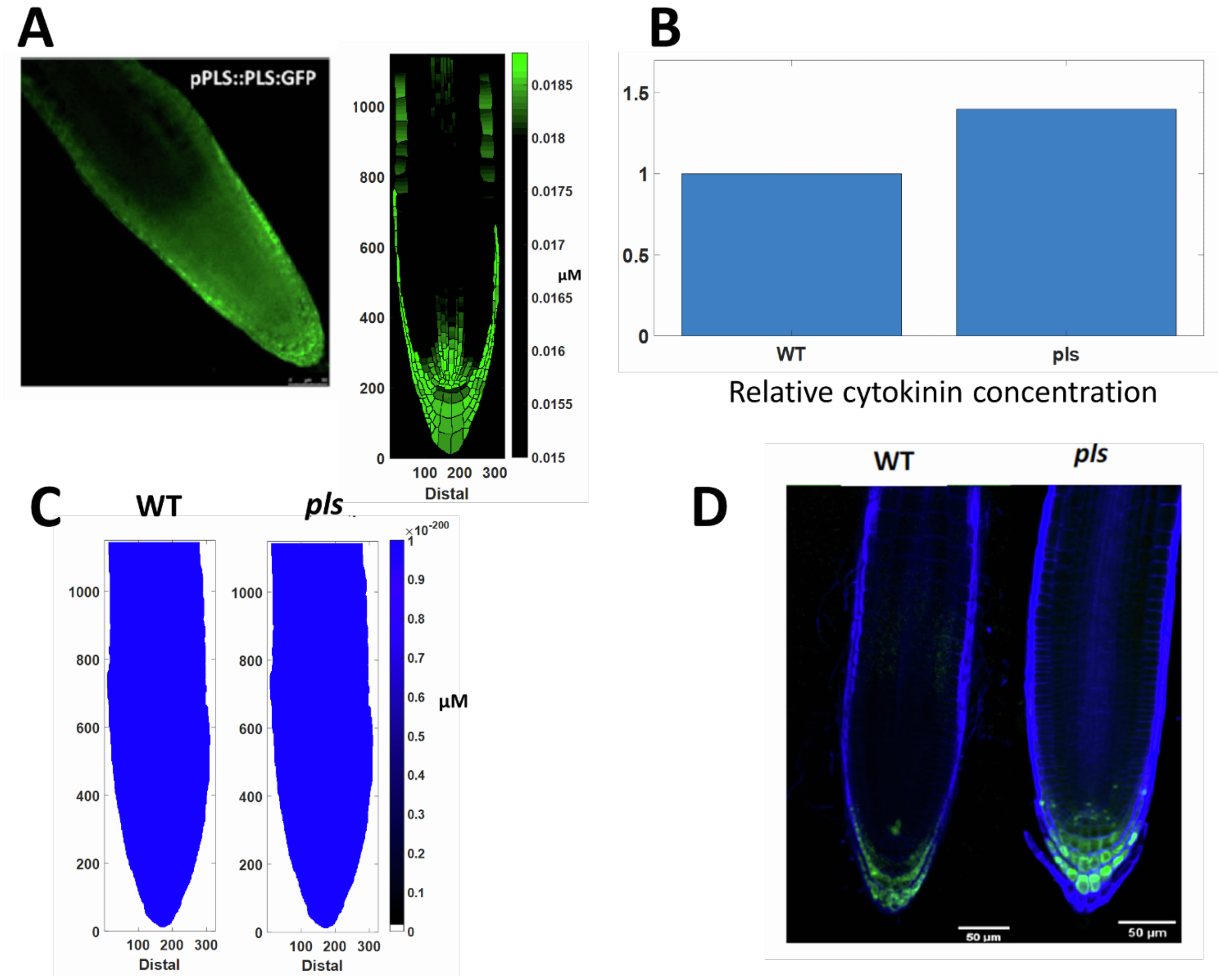

Figure S20. Same as Figure 7 in the main text, but in the absence of the regulation of cytokinin biosynthesis by auxin signalling (k18a=0).

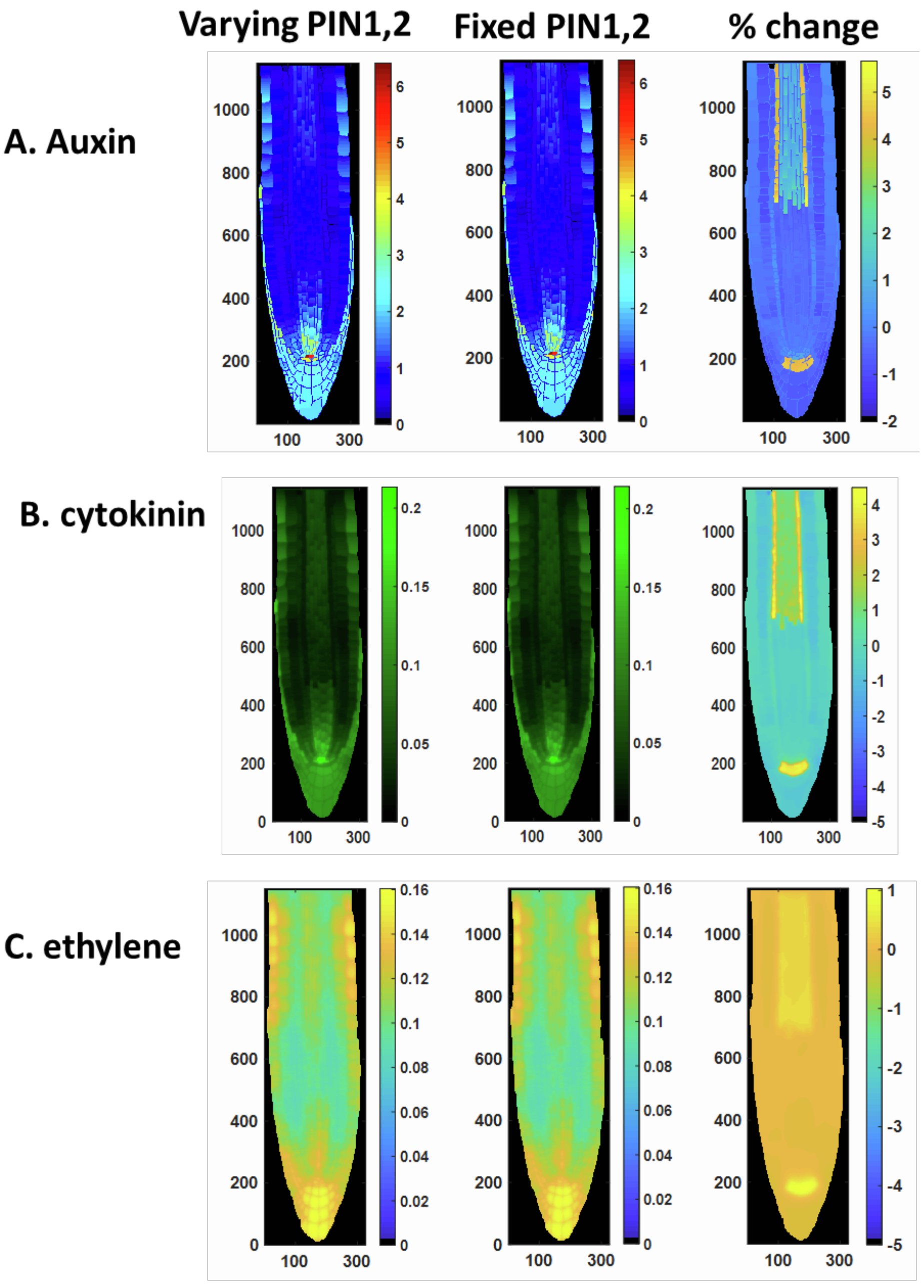

Figure S21. Role of changes in PIN1 and PIN2 concentration and patterning in *pin3* mutant. Figure 6 in the main text predicts change in PIN1 and PIN2 concentration and patterning in this mutant. Figure S21 shows that this change has a fine-tuning role in the concentration and patterning of auxin, cytokinin and ethylene. Varying PIN1 and PIN2 in this mutant is shown in Figure 6 in the main text. In 'Fixed PIN1,2' panels, PIN1 and PIN2 concentrations and patterning are fixed to WT levels.

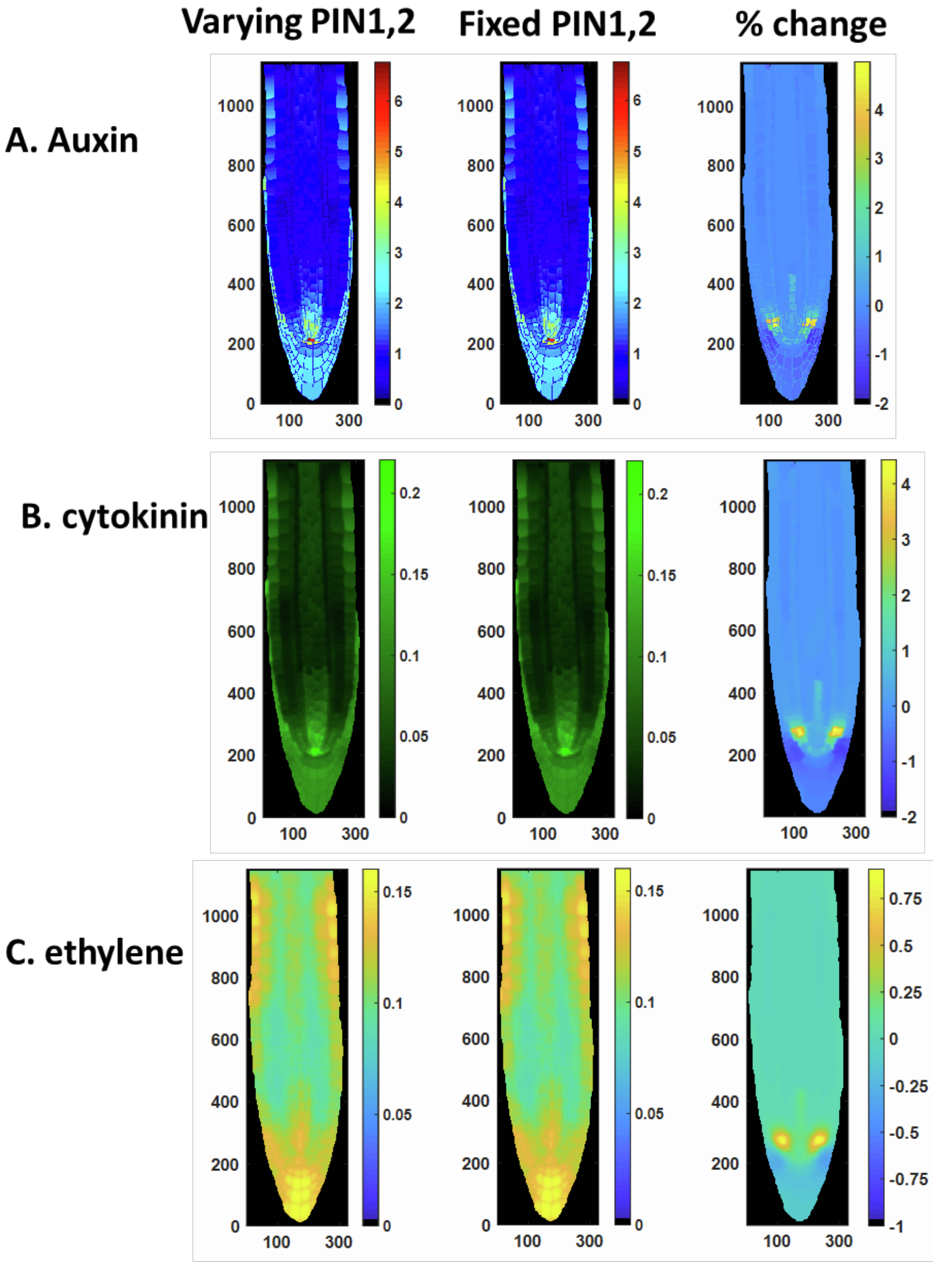

Figure S22. Role of changes in PIN1 and PIN2 concentration and patterning in *pin4* mutant. Figure 6 in the main text predicts change in PIN1 and PIN2 concentration and patterning in this mutant. Figure S22 shows that this change has a fine-tuning role in the concentration and patterning of auxin, cytokinin and ethylene. Varying PIN1 and PIN2 in this mutant is shown in Figure 6 in the main text. In 'Fixed PIN1,2' panels, PIN1 and PIN2 concentrations and patterning are fixed to WT levels.

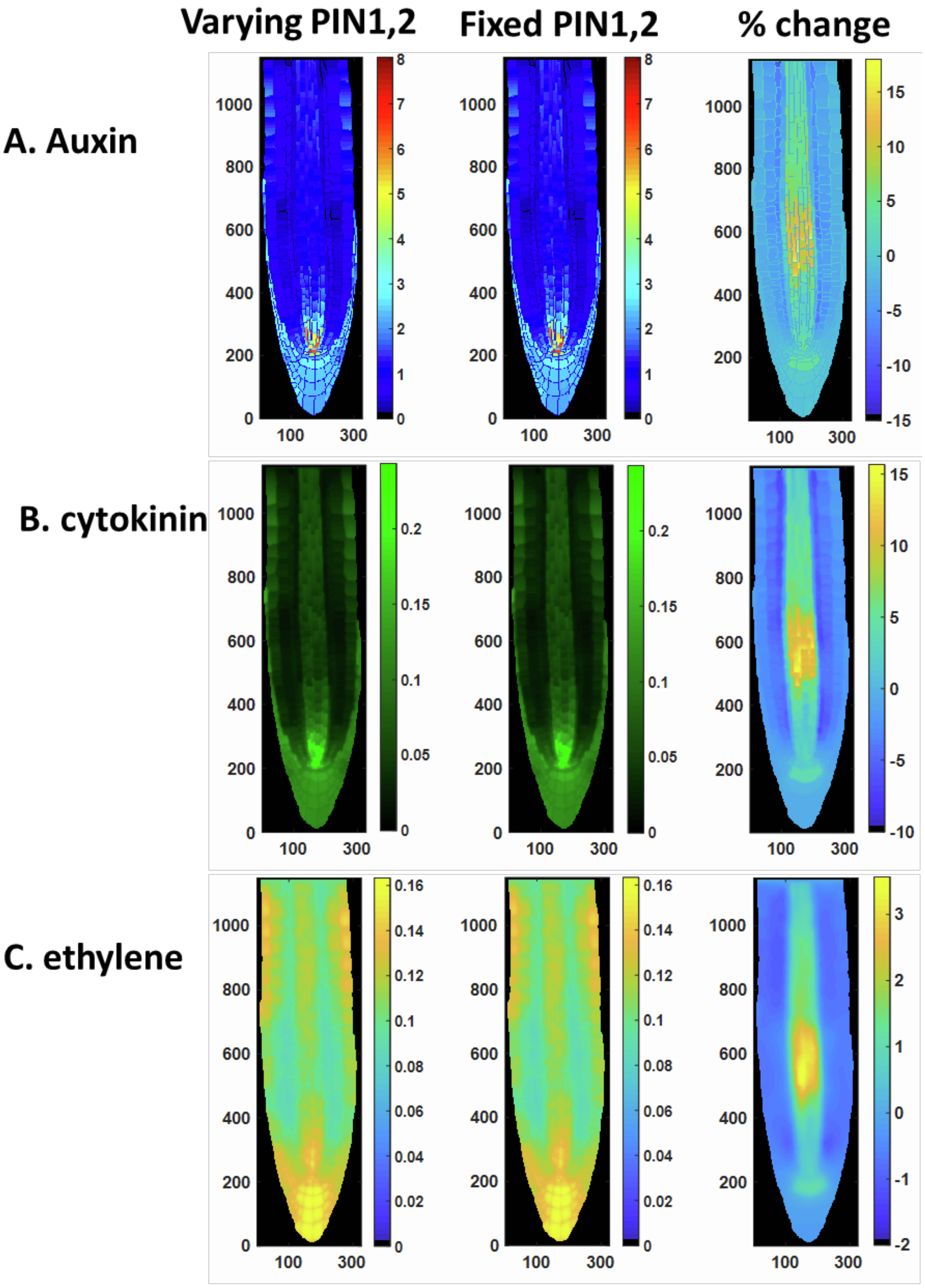

Figure S23. Role of changes in PIN1 and PIN2 concentration and patterning in *pin7* mutant. Figure 6 in the main text predicts change in PIN1 and PIN2 concentration and patterning in this mutant. Figure S22 shows that this change has a fine-tuning role in the concentration and patterning of auxin, cytokinin and ethylene. Varying PIN1 and PIN2 in this mutant is shown in Figure 6 in the main text. In 'Fixed PIN1,2' panels, PIN1 and PIN2 concentrations and patterning are fixed to WT levels.

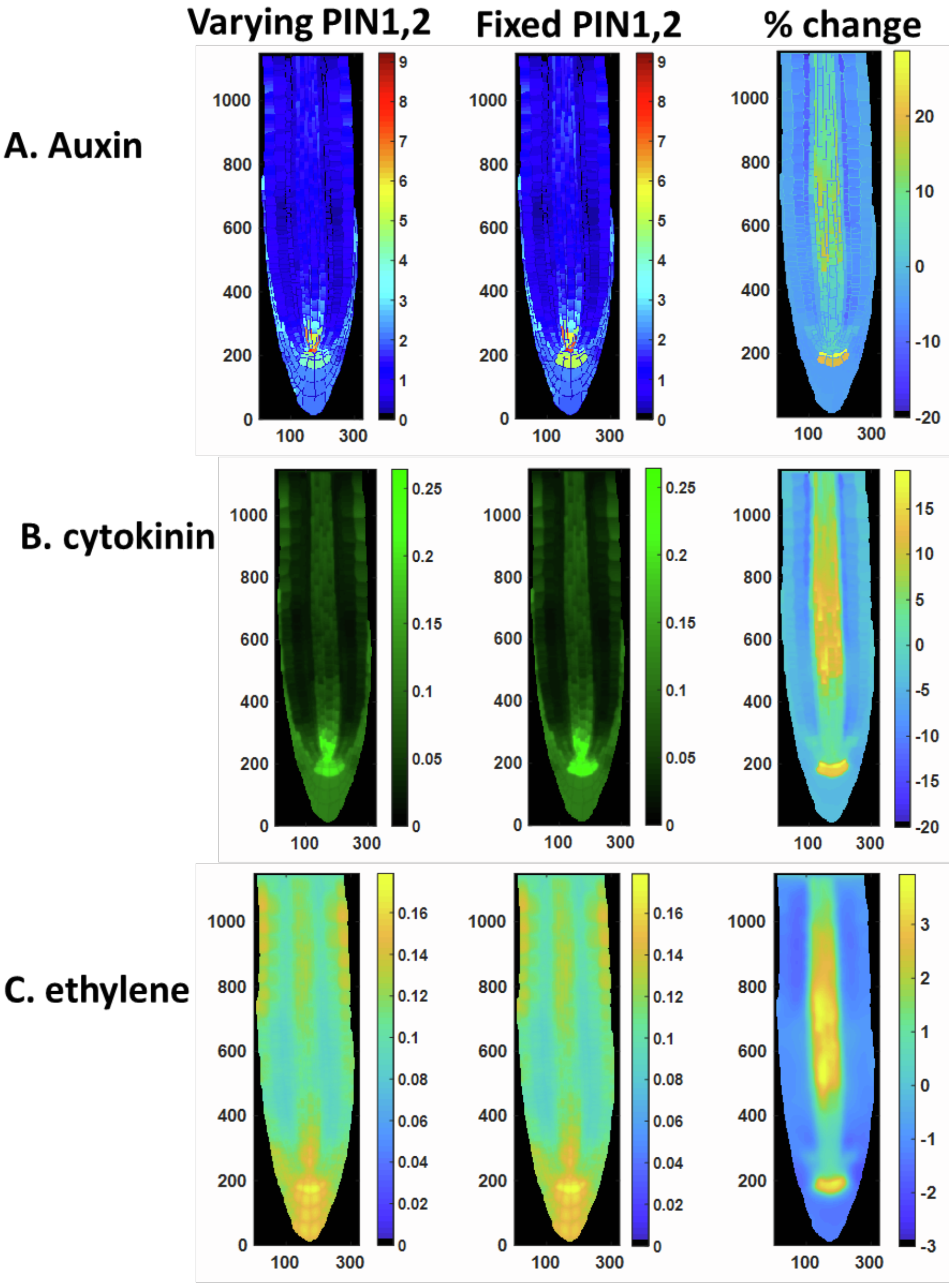

Figure S24. Role of changes in PIN1 and PIN2 concentration and patterning in *pin3,4,7* triple mutant. Figure 6 in the main text predicts change in PIN1 and PIN2 concentration and patterning in this mutant. Figure S24 shows that this change has a fine-tuning role in the concentration and patterning of auxin, cytokinin and ethylene. Varying PIN1 and PIN2 in this mutant is shown in Figure 6 in the main text. In 'Fixed PIN1,2' panels, PIN1 and PIN2 concentrations and patterning are fixed to WT levels.

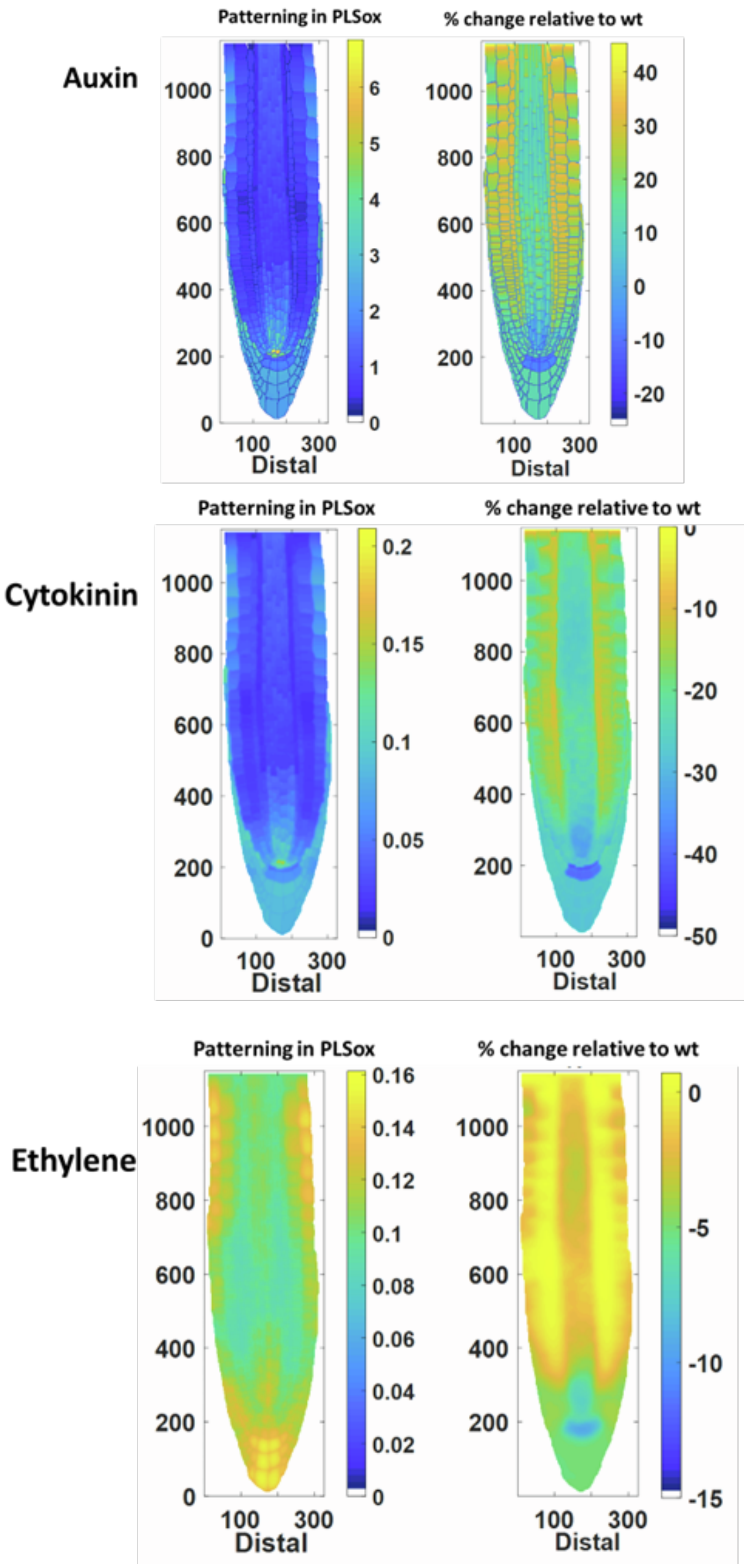

Figure S25. Role of PLS overexpressor in the concentration and patterning of auxin, cytokinin and ethylene. For the over expression of PLS (PLSox), k6 = 0.045 s^{-1}.

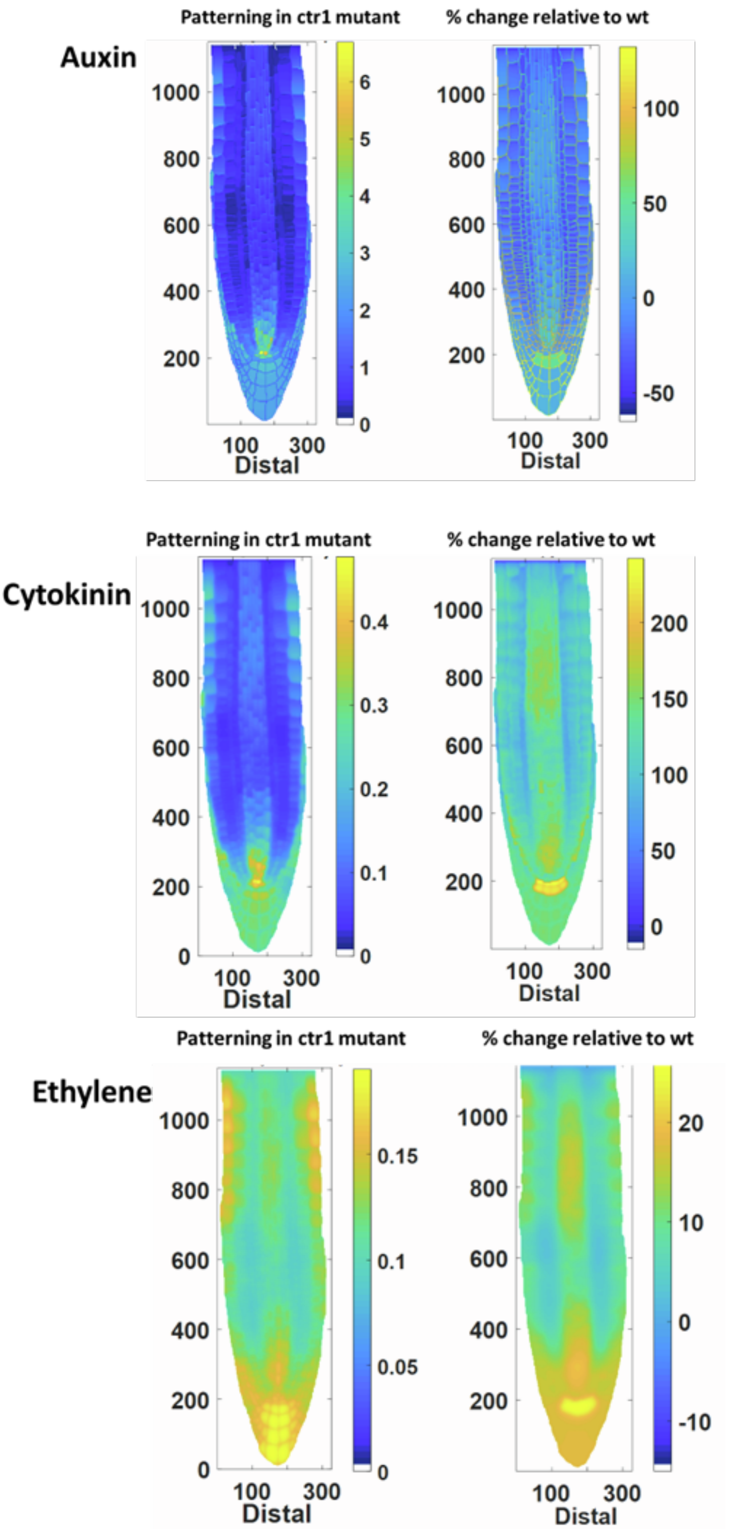

Figure S26. Role of *ctr1* mutant in the concentration and patterning of auxin, cytokinin and ethylene. For *ctr1* mutant, k14=0.0 $\mu M^{-1}s^{-1}$.

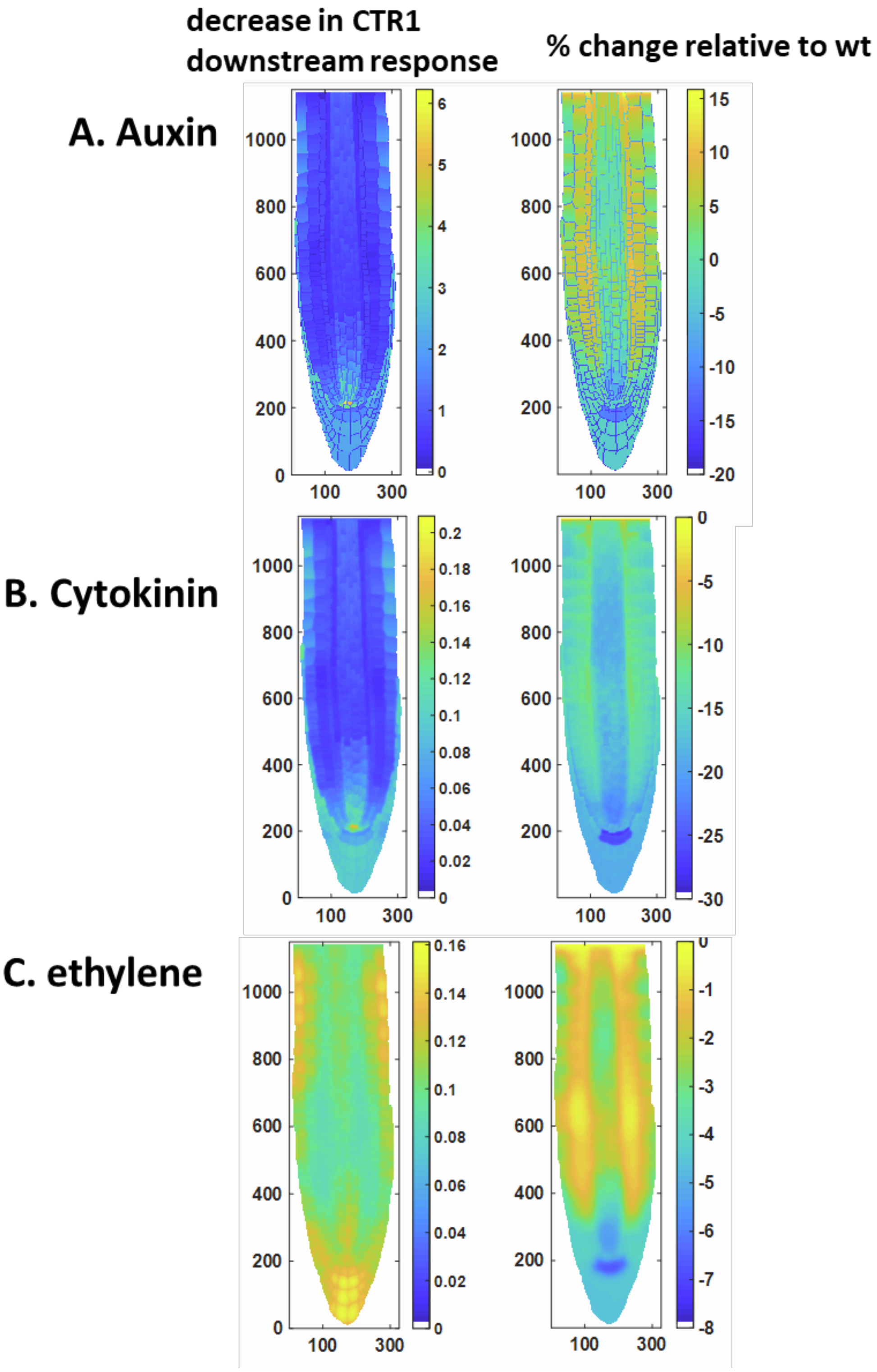

Figure S27. Role of decreasing CTR1 downstream response in the concentration and patterning of auxin, cytokinin and ethylene. Decreasing CTR1 downstream response is modelled using k16=0.28 μM s^{-1}.

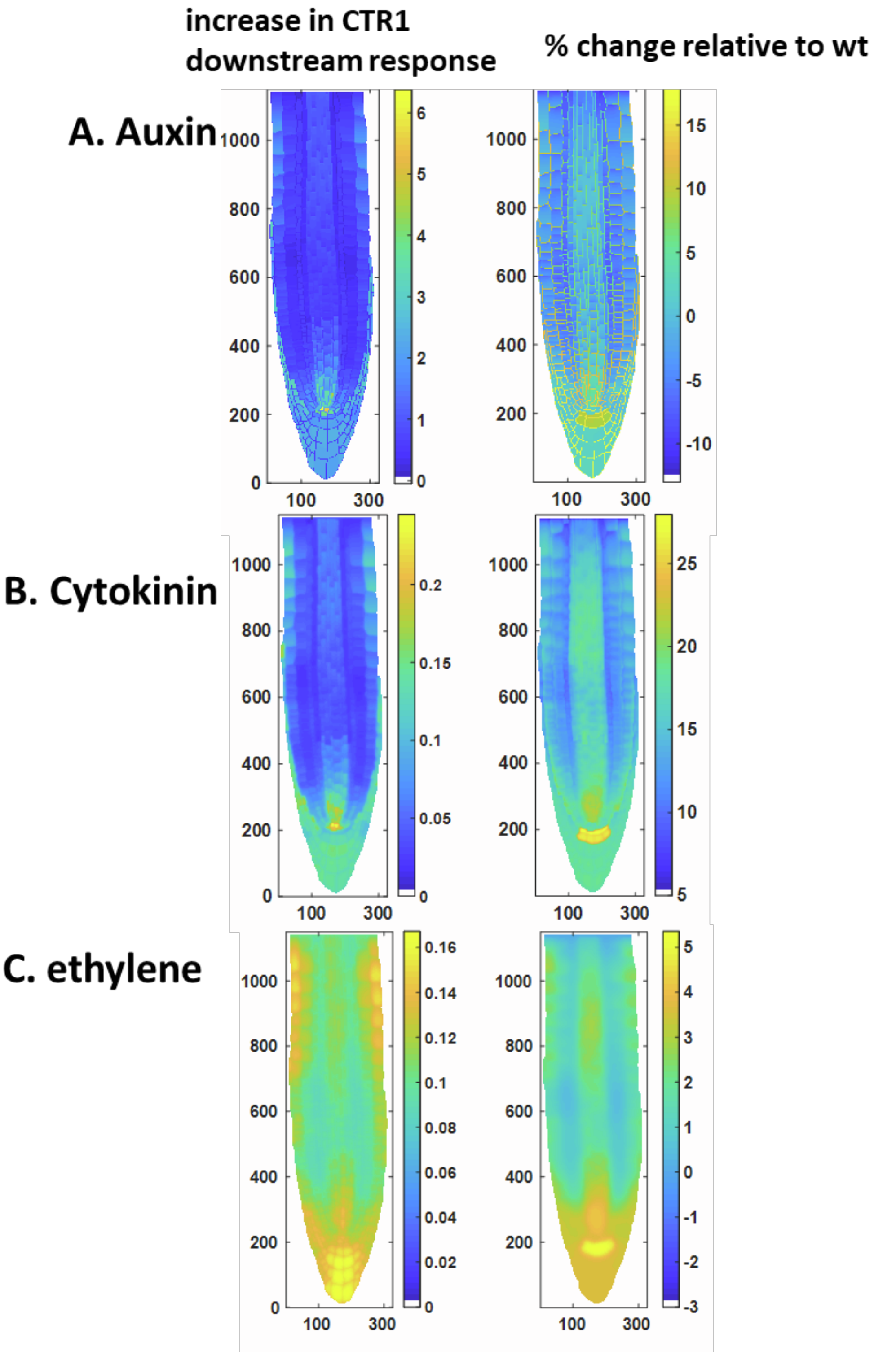

Figure S28. Role of increasing CTR1 downstream response in the concentration and patterning of auxin, cytokinin and ethylene. Increasing CTR1 downstream response is modelled using k16=0.32 µM s^{-1}.

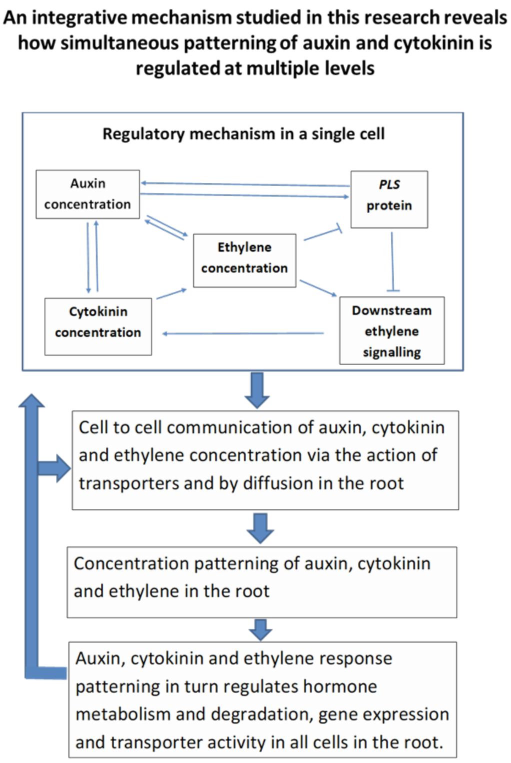

Figure S29. Schematic summary of the complex multi–level regulatory relationships for simultaneous auxin and cytokinin concentration patterning in the root.

Figure S29 describes an integrative mechanism reveals that simultaneous auxin and cytokinin concentration patterning emerges from multi–level regulation of auxin, cytokinin and ethylene in the root. Based on the experimental evidence discussed in the main text and summarised in Figure 1 in the main text, within each cell in the root there are several 2-way regulatory relationships, where auxin and cytokinin, auxin and ethylene, and auxin and the PLS protein all mutually promote the biosynthesis of the other. Ethylene signalling, which is promoted by cytokinin and inhibited by PLS protein (Casson et al. 2002; Chilley et al. 2006), also inhibits cytokinin degradation by inhibiting CKX through ARR2 and therefore promotes the accumulation of cytokinin (Figure 1 in the main text). Since cytokinin promotes ethylene biosynthesis (Vogel et al., 1998), cytokinin indirectly further promotes its own accumulation through the ethylene pathway. In contrast, cytokinin also inhibits its own accumulation by promoting auxin biosynthesis, which upregulates PLS, inhibiting ethylene signalling and promoting cytokinin degradation (Figure 1 in the main text). Thus, in each cell, auxin, cytokinin and ethylene mutually regulate their own concentrations at both metabolic and gene expression levels through multiple direct and indirect pathways.

In a root, auxin, cytokinin and ethylene all regulate cell-to-cell communications. In particular, they regulate activities of PINs and AUX1/LAX2,3 and they also regulate diffusion rate by regulating concentrations. Thus, a complex, multiple-level regulation exists for the functions of hormones in the root. This work focuses on three hormones (auxin, cytokinin, ethylene) and integrates metabolism, transport and gene expression into an integrative mechanism. We demonstrated such a mechanism is essential for elucidating the simultaneous patterning of auxin and cytokinin.
